# Tfh dysfunction underlies poor responses to HBV vaccination

**DOI:** 10.64898/2026.07.30.741822

**Published:** 2026-08-04

**Authors:** Han M. Chen, Elizabeth Severa, Xiaomin Yao, Tijaana Williams, Riddhimaa Sinha, Clarice Monteiro, Michael Tuen, Sam Barnett Dubensky, Sergei B. Koralov, Carla R. Nowosad, Derek A. Oldridge, Laura A. Vella, Anoma Nellore, Ramin Sedaghat Herati

**Affiliations:** 1. Department of Medicine, Division of Infectious Diseases and Immunology, NYU Grossman School of Medicine, New York CIty, NY.; 2. Medical Scientist Training Program, Perelman School of Medicine, University of Pennsylvania, Philadelphia, PA, USA.; 3. Department of Pathology, NYU Grossman School of Medicine, New York City, NY.; 4. Division of Infectious Diseases, Children’s Hospital of Philadelphia, Philadelphia, PA.; 5. Department of Microbiology, NYU Grossman School of Medicine, New York City, NY.

## Abstract

The primary goal of vaccination is to induce durable protection through generation of antigen-specific humoral immunity and cellular immune memory. Yet the same vaccine may elicit durable protection in some and less durable responses in others. Antigen-specific antibody responses arise from germinal centers (GCs), where T follicular helper (Tfh) cells help cognate B cells undergo somatic hypermutation (SHM), affinity maturation to generate humoral protection and cellular immune memory. In humans, however, the mechanisms linking GC dynamics to downstream antibody output remain incompletely characterized. To identify the molecular and cellular events that underlie differential vaccine responsiveness, we leveraged the known, pronounced variability in vaccine antigen-specific antibody concentration and durability observed after Hepatitis B virus (HBV) immunization. We longitudinally profiled 101 healthy adults undergoing either *de novo* (n=59) or booster (n=42) immunization. We then stratified participants into High (>100 mIU/mL) and Low (<100 mIU/mL) responder groups based on vaccine antigen-specific antibody responses. From this cohort, we obtained core needle biopsies (CNBx) of vaccine-draining lymph nodes from 10 participants (High Responder n=6 , Low Responder n=4), 10–21 days post-final vaccination and performed single-cell RNA sequencing with paired antigen receptor sequencing. Lymph node BCR repertoire analysis revealed convergent, semi-public clonotypes across unrelated participants with elevated SHM. Transcriptomic profiling of GC-B cells uncovered stark functional divergence between response groups, with High Responders showing robust activation across both dark zone (DZ) and light zone (LZ) compartments and enrichment of MYC and mTORC1 signaling. In contrast, Low Responders exhibited a contracted LZ compartment and a stalled GC B cell response. This divergence in B cell responses corresponded to changes in the T cell compartment. High Responders harbored an expanded Tfh subset marked by increased expression of CXCL13, ICOS, and GNG4, indicative of their GC localization and capacity to provide B cell help. Moreover, receptor-ligand analysis indicated that these GNG4+Tfh formed coordinated costimulatory networks capable of driving LZ GC-B cell licensing. Low Responders demonstrated a divergent program in which their GNG4+Tfh displayed aberrant inflammatory transcriptional activity. This inflammatory signaling was associated with reduced specificity of *BCL6* regulon activity despite maintained expression of canonical Tfh genes. Together, these findings reveal that variable HBV vaccine responsiveness was associated with transcriptional network alterations in the GNG4+Tfh subset, offering a molecular framework for understanding poor vaccine responses.

## One Sentence Summary

Lymph node Tfh dysfunction is associated with poor vaccine responses to Hepatitis B virus vaccination

## INTRODUCTION

The overarching goal of vaccination is to establish durable antigen-specific immune memory that provides protection through the generation of memory lymphocytes and long-lived plasma cells capable of sustained production of high-affinity protective antibodies. Optimal humoral responses depend on germinal centers (GCs), structures which are critical for affinity maturation, clonal selection, and differentiation of memory B cells (MBCs) and long lived plasma cells (1–5). Within GCs, T follicular helper cells (Tfh) provide essential help to cognate B cells, driving clonal selection and somatic hypermutation (5–7). Tfh subsets encompassing distinct functional states have been described in the setting of influenza vaccination in humans (7–10). Despite this knowledge, vaccination elicits variable humoral responses (11, 12). The intrinsic cellular and molecular programs that distinguish productive from unproductive vaccine responses in humans remain poorly understood.

An example vaccine known to elicit inconsistent humoral responses in immunocompetent adults is Hepatitis B virus (HBV) vaccination. Indeed, despite multiple doses, 5-10% of young, healthy adults fail to achieve seroprotective titers. Chronic inflammatory states, such as aging (13), obesity (14, 15), cigarette smoking (16), and comorbidities such as kidney disease (17) are further associated with impaired HBV vaccine responses. However, mechanisms underlying poor response to HBV vaccination are not well understood, though addition of CpG-based adjuvants improves responses (18–20). As lymph nodes are not routinely accessed following vaccination, the direct mechanisms underlying poor vaccine responses, even in otherwise healthy individuals, remain poorly understood and highlight a gap in knowledge which warrants additional investigation.

Elucidating the mechanisms that underpin differential vaccine responses to design more potent and durable vaccines therefore requires deep characterization of GC cellular dynamics at the site of response itself. Despite rich mechanistic insight from murine models, our understanding of human GC dynamics in the context of vaccination remains nascent. In humans, circulating Tfh (cTfh) and antibody secreting cells (ASCs) from peripheral blood following vaccination serve as accessible correlates of understanding GC dynamics during vaccine responses (21–27); however, these peripheral proxies offer limited resolution to the cellular dynamics within the lymph node, where GC reactions are initiated. To close this gap, recent studies deploying fine-needle aspiration (FNA) of vaccine-draining lymph nodes have demonstrated coordinated peripheral and GC responses in influenza (28–30) and SARS-CoV2 mRNA vaccinations (31–33), and impaired responses in immunocompromised kidney transplant patients (34). Together these studies establish the importance of GC responses in humans but provide incomplete mechanistic insights to underlying drivers of differential immune responses to the same vaccine antigen. Of note, one study in murine and human systems suggest inflammatory microenvironments can impair GC responses through differential Tfh responses (35). Thus, elucidating the mechanistic drivers of GC dysfunction remains a critical step towards understanding why some individuals mount robust responses while others respond poorly.

To better understand the link between GC responses and variability in humoral responses, we recruited ambulatory adults for longitudinal study of HBV vaccination (NCT04674462), including adults without prior history of HBV vaccination who received *de novo* vaccine series (n=59) and an additional cohort of HBV-immune adults who were given a single booster (n=42). Participants were stratified into High Responders and Low Responders based on final anti-HBV antibody titers. In addition, we performed an ultrasound-guided core needle biopsy (CNBx) of enlarged ipsilateral axillary lymph nodes in ten individuals in the 10-21 day period following the final vaccination. Immunofluorescence confirmed the presence of GCs. Via single-cell B cell receptor (BCR) analysis, we identified convergent GC-B cell responses including semi-public clonotypes across participants. High Responders exhibited hallmarks of robust GC responses relative to Low Responders, including more BCR mutations and proliferative capability in GC-B cells. Moreover, High Responders had GC-Tfh cells which maintained canonical costimulatory programs and robust T and B cell interaction networks required for B cell survival and affinity maturation, whereas Low Responders had fewer GC-Tfh as well as dysfunctional gene regulatory networks despite greater expression of some canonical Tfh genes. Together, these findings reaffirm the requirement for the coordinated engagement of GC B and T cells for productive responses and reinforce the need for further studies of Tfh to better understand suboptimal vaccine responses.

## RESULTS

### Heterogeneous humoral responses following Hepatitis B vaccination in adults

To interrogate the cellular and molecular mechanisms driving differential responsiveness to HBV vaccination, we prospectively enrolled 101 ambulatory participants across two main cohorts: a *de novo* vaccination cohort of HBV-naive adults and a booster cohort of adults with reported prior HBV immunization (NCT04674462; Fig. 1A, Fig. S1A). The median age of participants in the study was 55 (Supplemental Table 1). Individuals without prior HBV vaccination history (HBVnaive) were randomized in a 1:4 ratio to receive either two doses of CpG-adjuvanted (HBVnaive-CpG, n=13) or three doses of alum-adjuvanted vaccine (HBVnaive-Alum, n=45), whereas those with prior HBV vaccination (HBVimmune, n=42) received a single alum-adjuvanted dose (Fig. 1A, Fig. S1A). As per package insert guidelines, HBVnaive-Alum participants received vaccinations at approximately 0, 4 and 24 weeks after study entry, whereas HBVnaive-CpG received vaccinations at 0 and 4 weeks. For all three study arms, we collected blood at the time of vaccination (w0), one week after each vaccination (w1) and four weeks after vaccination (w4), facilitating longitudinal analysis of antibody kinetics across successive vaccinations. Across three study arms, we collected 508 blood samples from the 101 participants. Additionally, participants who consented underwent screening for enlarged ipsilateral axillary lymph nodes amenable to core needle biopsy (CNBx) 10 to 21 days after final vaccination, an opportune window for capturing GC dynamics following immunization (32, 36–38).

To track humoral responses to HBV surface antigen (HBsAg), we quantified circulating HBsAg-specific antibodies (HBsAb) by ELISA. In each of the three study arms, we observed overall HBsAb increase following the vaccine series, with a geometric mean increase of 2.8-fold for the HBVimmune group at end of study, 16-fold for HBVnaive-Alum and 6.9-fold for HBVnaive-CpG by one month after the final vaccination relative to baseline (Fig. 1B, Fig. S1B). Similar distribution of endpoint titers was observed between the HBVimmune and HBVnaive-Alum groups (p>0.05, n=42 vs n=45, one-way ANOVA with Tukey’s post-test; Fig. 1C). Aggregate end of study geometric mean HBsAb was 97.1 mIU/mL, with group-level means of 89.2 mIU/mL (HBVimmune), 101.0 mIU/mL (HBVnaive-Alum), and 122.0 mIU/mL (HBVnaive-CpG). Thus, we stratified participants into High (>100 mIU/mL) and Low (<100 mIU/mL) Responders to understand variability in the humoral response, rather than employ the traditionally-used seroprotective threshold of 10 mIU/mL (39). Although titers did not differ at baseline between High and Low Responder cohorts, these cohorts did differ in the pharmacodynamic response to immunization. In the HBVnaive-Alum arm, antibody titers in Low Responders notably did not rise after the second vaccination and also demonstrated a limited response to the third vaccination (P=3.2e-4, linear mixed effects (LME) interaction, Fig. 1D). Moreover, we observed a similar stalled pharmacodynamic response in HBVimmune (overall: P=2.5e-9, LME interaction), and to a lesser extent, in the CpG-adjuvanted arm (p=3.96e-02, LME interaction, Fig. S1C). CpG-adjuvanted vaccination resulted in a high proportion of participants achieving >100 mIU/mL though this proportion was not different from the HBVnaive-Alum cohort (p=0.73, n=10 vs n=45, Fisher’s Exact). Moreover, High and Low Responder cohorts were not associated with differences in age, sex, body mass index, or smoking status (multivariate linear regression, Fig. S1D). Overall, these results establish a well-characterized clinical cohort in which vaccine regimen and pre-existing HBV immunity shape the magnitude of humoral responses, but antibody titer stratification into High and Low Responders revealed pharmacodynamic differences in response kinetics that were not explained by demographic or clinical variables alone.

### Ipsilateral lymph node enlargement following vaccination

To uncover immune mechanisms associated with humoral responses, we turned to direct lymph node evaluation after vaccination. Of the 86 individuals offered CNBx, 72 (83.7%) consented to ultrasound screening, of whom 10 (13.9%) had lymph nodes amenable to ipsilateral ultrasound-guided biopsy with an average of 15.6 (SD 3.6) days after the final vaccination visit. Ultrasound measurements of lymph nodes were a mean area of 0.79 cm^2^, long axis length of 1.43 cm and cortex thickness of 0.29 cm (Fig. 1E, Fig. S1E), measurements similar to those reported following SARS-CoV2 vaccination (40, 41). We considered that participants with lymphadenopathy might differ from participants without lymphadenopathy. However, CNBx participants did not have uniformly strong humoral responses and their distribution of antibody responses was similar to that of the overall cohorts (Fig. 1C). The CNBx subgroup also reflected the overall study age distribution (N.S., Mann-Whitney; Fig. S1F). Although addition of CpG adjuvant improves antibody responses (19, 42), lymph nodes were not consistently enlarged in these individuals either, as the rates of CNBx were 16.7% (n=1) for HBVnaive-CpG vs 14.3% (n=3) for HBVnaive-Alum, and 24% (n=6) for HBVimmune (Fig. S1G). We also considered participants’ recent vaccine history, as prolonged GC reactions following vaccination are described (43), but only one participant had had any recorded vaccination in the 6 months prior to study entry, and laterality could not be established. Thus, the CNBx subset appeared broadly representative of the larger cohort, although we cannot exclude other differences between individuals with and without lymphadenopathy.

We next evaluated the biopsy specimens histologically. For each of the 10 participants, one core was formalin-fixed and paraffin-embedded (FFPE) for histology while remaining cores were mechanically dissociated into cell suspensions and frozen for future use. Of ten biopsy sections, six contained lymphoid tissue on hematoxylin and eosin staining. To determine presence of germinal centers, immunofluorescence staining for Ki67, IgD, and PD-1 was first optimized in unrelated, deidentified tonsils. Then, we evaluated CNBx FFPE tissue sections, finding visible germinal centers in 4 of 10 sections. These GCs displayed the expected polarization of Ki67 and PD-1 though were smaller than tonsil GCs (p<0.05, Mann-Whitney with Benjamini-Hochberg correction; Fig. 1F–G). We did not identify GCs in all specimens, which may reflect sampling error or lymphadenopathy unrelated to vaccination as only one biopsy core per participant was FFPE preserved and evaluated. Still, these data directly demonstrate the presence of GCs in at least some ipsilateral lymph nodes following HBV vaccination.

### Lymph node biopsies reveal active and convergent germinal centers

To interrogate the cellular and clonal dynamics driving these divergent humoral responses described above, we next performed single-cell RNA sequencing of dissociated viable cells from all 10 CNBx participants (Fig. S2A). We recovered a total of 22,037 B cells across all participants. Given the dependence of longitudinal antibody responses, we focused our analysis on memory B and plasma cell differentiation. Unsupervised clustering and uniform manifold approximation and projection (UMAP) visualization resolved six distinct B cell populations as determined by differential gene expression and as previously described (28, 44, 45): dark zone (DZ) and light zone (LZ) GC B cells, plasma cells, FCRL5^hi^, naive B, and memory B cells (Supplemental Table 2; Fig. 2A–B, Fig. S2B–C). B cell receptor analysis revealed that GC-B cell and plasma cell clusters had a higher proportion of mutated IGHV when compared to non-GC clusters (P<0.001 for all comparisons, Wilcoxon rank-sum test with Benjamini-Hochberg adjustment; Fig. 2E), further supporting annotation. Notably, GC-B cell proportions varied between participants (Fig. S2D) and did not differ between immune and naive individuals (Fig. S2E).

We next evaluated clonal relationships between B cell subsets based on BCR analysis. Within individual repertoires, expanded clones represented a minority of the total captured B cell pool (Fig. 2C). As expected, these clonal expansions were predominantly localized within the GC compared to resting or non-GC compartments (Fig. 2D–F). Indeed, we observed clonal population enrichment for light zone (LZ) and dark zone (DZ) phenotypes, whereas unexpanded singletons mapped almost exclusively to non-GC B cell states (Fig. 2F). To resolve the ongoing somatic hypermutation (SHM) and selection pressures shaping GC populations, we reconstructed phylogenetic lineage trees for individual expanded clones using single nucleotide maximum likelihood (Fig. 2G). Expanded clones were predominantly LZ and DZ B cells, and were composed of IgG and IgM isotypes (Fig. 2F–G). Together these data suggests a GC and targeted response.

The HBV vaccine encodes HBsAg, which encodes the highly conserved “a” determinant that is shared across HBV genotypes and is the target of protective antibody responses (46, 47). We considered whether HBV vaccination may induce a targeted repertoire against this determinant that may lead to emergence of semi-public clonotypes. To assess this, we considered BCR across individuals, defined by identical VH and JH gene usage and junction length, and whose HCDR3 amino acid sequence similarity was at least 85% (Fig. 2H). We found 129 total clonotypes meeting these criteria, including 93 (72%) which were present in at least two unique participants and 47 (36%) which were shared by three or more individuals (Fig. 2H–L, S2F), suggesting convergent clonotypic selection. Indeed, these convergent clonotypes were predominantly composed of GC B cells and plasma cells (Fig. 2M). As with GC responses following vaccination (32, 48, 49), IgG isotype dominated convergent repertoires followed by IgM (Fig. S2G). To determine if BCR responses are HBV-specific, we compared our CNBx repertoires to previously reported HBV-specific antibody clonotypes (50–52). We identified 10 clonotypes across 5 participants with high HCDR3 similarity (>80% amino acid similarity) to clonotypes described in the literature (50–52) including semi-public clonotypes shared across multiple individuals (Supplemental Table 3).

We next evaluated whether these convergent clonotypes had undergone SHM and clonal expansion. To address this, we assigned all clones within a convergent clonotype as shared. Because a single convergent clonotype represents only one sequence within a larger clonal lineage, we mapped each convergent clonotype onto its corresponding clonal lineage tree and designated any clonal tree lineage containing a convergent clonotype as shared. Indeed, convergent BCR were enriched for GC-B cells relative to non-shared sequences (Fig. 2N). Moreover, relative to non-convergent repertoire, convergent lineages exhibited greater mutational burden in *IGHV* (Mann-Whitney, P = 1.1e-7; Fig. 2O), and longer HCDR3 sequences (Mann-Whitney, P=2.1e-4; Fig. 2P). Furthermore, consistent with repertoire enrichment following vaccination and viral exposure, shared repertoire harbored greater IgG isotype representation, and specifically, enrichment of IGHG1 (Fisher’s exact, P =0.014; Fig. 2Q). Consistent with skewing of the repertoire following vaccination and viral exposure (53, 54), VH gene usage was enriched for *IGHV3-23*, *IGHV1-69*, and *IGHV4-34* in convergent repertoire relative to non-shared (Fig. 2R), of which *IGHV3-23* (Fig. S2H) has been previously reported enriched in antigen specific HBV vaccine responses (50). Together, these data suggest convergent BCR responses to HBV vaccination across individuals, likely driven by germinal center selection.

### Germinal center B cell activity and transcriptional programs underlie robust humoral responses

Despite the convergence of BCR responses in the CNBx subgroup, vaccine titers were nonetheless variable (Fig. 1C). We therefore sought to identify the cellular and transcriptional determinants of this divergent antibody output, using the High and Low Responder stratification described in Fig. 1D. We considered whether GC function was equivalent between the two cohorts, as productive GC would be expected to accumulate mutations. DZ and LZ GC-B cells from High Responders were more mutated relative to germline than those from Low Responders, consistent with greater iterative cycling (Fig. 3A, S3A–B). Complementing this, phylogenetic analysis of clonal trees further revealed an association between maximum germline divergence and aggregate tree length that differed by responder category, with a steeper accumulation of mutation (germline divergence) per unit tree length in High than Low Responders (robust linear regression, stratified bootstrap; P = 0.031; Fig. S3C), suggesting greater diversity in the clonal response and coexistence of parallel affinity trajectories that may lead to affinity-promoting mutations (55, 56). These data suggest more robust GC activity may contribute to the strong humoral responses in High Responders.

To identify underlying transcriptional programs directing vaccine responsiveness, we performed differential gene expression analysis of GC B cell subsets (Fig. 3B–C, Supplemental Table 4). In High Responders, DZ GC-B cells had greater expression of heat shock protein family members (*HSP90AA1, HSPH1, HSP90AB1, HSPD1*) as well as co-chaperones (*DNAJC15, DNAJA1, DNAJB1*), which may support *BCL6* in the DZ compartment (57). In the LZ compartment, High Responders similarly upregulated heat shock protein family members (*HSP90AA1, HSPH1, HSPA1B, HSP90AB1, HSPD1, HSPA8, HSPE1*) and DNAJ protein family members (*DNAJA1, DNAJB1, DNAJB6*), while Low Responders were enriched for *SELL, SYK, FCRL1*, and *JCHAIN*, a transcriptional profile more consistent with early activation than committed GC programming, as predicted (58). Together, these DZ and LZ gene expression differences delineate a divergence in GC-B cells where High Responders are transcriptionally poised for activity and Low Responders have stalled at preGC or quiescent states.

Pathway analyses confirmed inference from individual transcripts. The DZ GC-B cells from High Responders had increased pathway activity for MYC targets, NFκB signaling, mTORC1 signaling, and G2M checkpoint, indicative of more active metabolic engagement and productive GC cell cycling (Fig. 3D), consistent with the literature (59). The LZ compartment exhibited a divergence between High and Low Responders (Fig. 3E). High Responder LZ GC-B cells were also differentially enriched for TNF/NFκB signaling, Inflammatory response, mTORC1 signaling, and MYC target gene sets, which likely reflect non-canonical NFκB signaling, which is downstream of CD40 and drives MYC upregulation in the LZ B cells (60). However, Low Responders also had enrichment of the P53 Pathway, which may indicate cell cycle arrest, as well as IL2/STAT5 signaling, which is antagonistic to Bcl6 expression (61). Similar patterns of differences were seen in Plasma Cells (Fig. S3D). Taken together, these data suggest that LZ B cells from High Responders were highly receptive to positive selection signals from Tfh, whereas Low Responder LZ B-cells were enriched for pathways indicating a failure to undergo this costimulatory transition, and instead remained in a metabolically quiescent or stalled state.

Having identified gene and pathway differential expression divergence, we next sought to determine whether these differences were underpinned by distinct regulatory programs. We inferred transcription factor activity networks using decoupleR within LZ and DZ compartments independently and stratified by High and Low Responder groups. High Responders displayed a consistent proteostatic and proliferative signature spanning both compartments, marked by heightened activity of heat shock and unfolded protein response regulons *HSF1*, *HSF2*, and *DDIT3*, alongside *MYC* (Fig. 3F). This proteostatic program was pronounced in the LZ, where High Responders additionally exhibited elevated ATF4 and *CIITA* activity, consistent with a coordinated transition toward productive and active responses including antigen presentation and antibody production. In contrast, Low Responders failed to sustain these proliferative programs and instead exhibited a subdued profile more consistent with early activation. In the DZ, this was characterized by elevated Hippo activity (*YAP1*, *WWTR1*) (62, 63) paired with reduced *PPARGC1B* and *HDAC3* activity. In the LZ, Low Responders aberrantly sustained B cell identity programs (*PAX5, SPI1, SPIB*, and *IKZF3*). Together, these data support a model where High Responders progress through a robust GC program maintaining B cell identity during DZ proliferation, then efficiently dismantled during LZ selection, whereas Low Responders fail to execute this transition at either checkpoint.

To corroborate these inferred regulatory landscapes, we utilized single-cell regulatory network inference and clustering (SCENIC) as an orthogonal regulon analysis (64, 65) (Fig. 3G, Supplemental Table 5). Within the LZ compartment, High Responders demonstrated dominant *RELB* and *NFκB2* activity, drivers of the non-canonical NFκB pathway activated downstream of CD40. Stress responsive regulons *ATF4, ATF3, CEBPB,* and *CEBPG* reflected productive GC-B cell responses, while *IRF1* and *IRF9* activity aligned with heightened inflammatory and interferon signaling observed at the pathway level. Conversely, Low Responder LZ GC-B cells demonstrated dominant *IRF2, PAX5, STAT2,* and *BCL6* regulon activity. Of note, this profile lacked active GC selection signatures prominent in High Responders and instead reflected a diminished state marked by persistence of cell identity (*PAX5, BCL6*) alongside a distinct repressed inflammation character (*IRF2*).

Finally, we evaluated whether the frequency of the GC B cell subsets was different between these cohorts. Indeed, miloR differential abundance analysis demonstrated LZ GC-B cell enrichment in High Responders relative to Low Responders (Fig. 3H), supportive of more productive Tfh driven selection. Together, these transcriptional and regulatory analyses support a model in which High Responders have more productive GCs, while Low Responders show signs of stalled selection governed by Tfh.

### T follicular helper subsets are spatially segregated

The enrichment and active state of LZ GC-B cells in High Responders suggested that these B cells were undergoing more robust interaction with LZ-positioned Tfh and identified the LZ region and T cell help as most relevant for investigating determinants of differential humoral response. To understand whether the Tfh programs differ between response groups, we first performed unsupervised clustering and UMAP visualization of the CD4+ T cells recovered from lymph node biopsies post-vaccination. The cluster analysis revealed distinct populations of naive, memory, regulatory, and Tfh cells (Fig. 4A, Fig. S4A, Supplemental Table 2). To evaluate for the presence of HBV-specific CD4+ T cells, we mapped HBV-specific CD4+ T cell clonotypes by cross-referencing CDR3 sequences with known HBV epitopes from a study of chronic HBV infection (66) in the IEDB database. In total, we identified 44 matching clonotypes across all CD4+ T cells (P=8.1e-96, Fisher’s Exact test) including 4 in the Tfh cluster (P=7.4e-6, Fisher’s Exact test, Fig. 4B, Fig. S4B), indicating the presence of antigen-specific lymphocytes in the Tfh cluster (66).

We then specifically focused on the Tfh compartment, which we identified by unsupervised clustering of CD4+ T cells and by expression of canonical markers including *CXCR5*, *BCL6*, and *CXCL13*. We performed further subclustering of these 2,995 Tfh cells, as it has become increasingly recognized that Tfh subsets may have distinct properties (8, 35, 67). Subclustering of the Tfh cluster revealed six distinct subsets (Fig. 4C–D, Fig. S4C). These subclusters were named according to gene expression pattern (Fig. 4C, 4E, S4D). The subset associated with the classical germinal center Tfh state was designated the GNG4 subset and was characterized by robust co-expression of *CXCL13*, *POU2AF1*, *TOX2*, *BCL6*, and *CXCR5*. Another subset, termed the GPR183 subset, was marked by the expression of *GPR183*, *CD69*, *JUNB*, and *FOS*. We also identified a TNFR2 subset expressing early activation genes including *LAG3*, *CD38*, *TNFRSF1B*, *IL10*, and *PRDM1*; an IL7R subset indicative of a quiescent or memory-like state (*IL7R*, *KLF2*, *TXNIP*); a VIM subset characterized by structural and migratory markers (*PTMS*, *S1PR1*, *CCR7*); and an HSP subset enriched for stress response genes (*HSPA1A*, *HSPA1B*). Although all subsets had similar Shannon’s entropy, the GNG4+ subcluster consisted of an elevated number of unique clonotypes (Fig. 4F, Fig. S4E). TCR overlap analysis showed substantial sharing of clonotypes between the GNG4, TNFR2, and GPR183 states (Fig. 4G), suggesting potential transitions between these functional states within the lymph node. Furthermore, clonotypic expansion, which was defined as observation of two or more clones of the same clonotype, was most visible in the GNG4 and TNFR2 subsets (Fig. 4H). Finally, to infer potential developmental relationships between these Tfh subsets, we performed unsupervised trajectory analysis using Slingshot (68). Pseudotime ordering arranged the cells along a continuous differentiation trajectory, anchoring the root in the less differentiated, memory-like TNFR2 subset (Fig. 4I–J). Other subsets such as GPR183 and IL7R had intermediate pseudotime values, whereas the GNG4 subset had the greatest pseudotime values and may reflect progression towards the GC-Tfh state.

To delineate the regulatory networks orchestrating these diverse transcriptional profiles, we performed SCENIC (64, 65) (Supplemental Table 5). By evaluating transcription factor binding motifs alongside co-expression modules, we identified unique regulons driving the specialization of each Tfh state (Fig. 4K, Figure S4F–G). The GNG4 subset was governed by an expected regulatory program driven by master transcription factors including *POU2AF1*, *BCL6*, *CEBPA*, *TBX21*, and *BATF*, in accordance with prior studies (69). In stark contrast, the GPR183 subset lacked these definitive GC regulators and was instead dominated by a marked activation of the AP-1 complex network, exhibiting high specific activity for *JUN*, *JUND*, *FOS*, *FOSB*, *EGR1*, *JUNB*, and *HIF1A*, which suggests the GPR183 subset represents a highly activated, early-primed state. In contrast, the TNFR2 subset was associated with transcription factors associated with terminal differentiation and activation-induced dampening, heavily utilizing *PRDM1* (Blimp-1), *CREB3L2*, and *BHLHE40* regulons which are known to regulate Tfh programs (70, 71). Conversely, the *IL7R* subset was actively maintained by canonical T cell memory and stemness regulators, primarily *TCF7*, *LEF1*, and *KLF2* which also have been associated with restraint of the Tfh program (72). Finally, the stress-associated *HSP* subset was transcriptionally isolated by its reliance on heat-shock factors, specifically *HSF1*, *HSF2*, and *CEBPB*. Together, these distinct regulon architectures perfectly mirror the phenotypic partitioning of the cells, detailing the precise gene regulatory networks required for stepwise CD4+ T cell differentiation into mature HBV-specific GC Tfh cells.

We next sought to identify the physical location of Tfh transcriptomic subsets within the intact lymphoid architecture to determine which would be most likely to be involved in the germinal center reaction. We mapped the transcriptional signatures of our Tfh subsets onto a reference dataset of a human tonsil with spatial clustering generated using Visium HD (Fig. 4L–N, Fig. S4H–J). One of these spatial clusters, Cluster 8, was identified as the mature germinal center core based on dense *BCL6* expression. Quantitative composition analysis of the Cluster 8 spatial bins revealed that the *GNG4* signature constituted nearly all of the Tfh signal within the GC niche. Conversely, the TNFR2 and GPR183 subsets mapped predominantly to the extrafollicular space and the T-B border regions, thereby corroborating their potential roles as activated and pre-Tfh transitional states in the setting of immature GCs.

Based on the evident physical proximity of the GNG4+Tfh with LZ-B cells, we evaluated receptor-ligand interactions of these two subsets using LIANA (73). The GNG4+Tfh subset was the only Tfh subset that demonstrated *CXCL13-CXCR5* interaction (Fig. 4O), consistent with prior literature (74), as well as interactions such as *NTRK5-PTPRS* known to be regulated by STAT3 (75). Together, these data support the notion that the GNG4+Tfh subset is the primary subset interacting with LZ-B cells in the germinal center.

### GNG4+Tfh cell expansion is a hallmark of robust humoral responses to HBV vaccination

Having established a molecularly-defined map of the CD4+ T cell landscape and defined discrete Tfh functional states, we next sought to determine how these subsets correlated with vaccination outcomes. We first evaluated compositional shifts in the Tfh compartment relative to vaccine response. Using Cohen’s *d* effect size calculation for each Tfh subset, the strongest association between subset frequency and antibody titer was in the GNG4+Tfh subset (Fig. 5A, S5A). Indeed, the GNG4+Tfh subset was over 10.1-fold more likely to be associated with high response than low response, followed by the TNFR2 subset at 5.2-fold. We also performed miloR differential abundance testing to evaluate the different clusters, which demonstrated that the GNG4 subset was most reliably associated with high antibody responses, followed by the GPR183 subset (Fig. 5B–C, S5B). Notably, several subsets were associated with Low Responders, such as the HSP and IL7R subsets, which may reflect a failure to fully engage mature, follicle-resident helper cells. We thus focused on the GNG4+Tfh subset given its spatial position in the GC and its effect size.

Although the abundance of GNG4+Tfh cells was associated with high HBsAb titers, we also considered that qualitative, cell-intrinsic functional deficits might also contribute to suboptimal vaccine responses. To address this, we performed differential gene expression and pathway enrichment analyses restricted to the GNG4+Tfh compartment, comparing cells derived from High versus Low Responders (Fig. 5D, S5C, Supplemental Table 4). The top genes associated with GNG4+Tfh from High Responders included *BACH2* and *REL*, whereas the top genes in Low Responders included *PDCD1*, *JUN*, and *TXNIP*. We then evaluated per-cell gene expression for genes characteristically associated with Tfh function, which demonstrated greater expression of *IL21*, *CD40LG*, and *POU2AF1* in GNG4+Tfh from Low Responders (Fig. 5E). We next modeled intercellular ligand-receptor interactions to determine if the pattern of differential expression of GNG4+Tfh was associated with altered LZ B cell interactions (Fig. 5F, Fig. S5D–E, Supplemental Table 7), recognizing that the primary function of GC Tfh cells is to provide cognate help to B cells. Differential interaction network modeling using LIANA (76) to evaluate GNG4+Tfh and LZ B cells demonstrated enrichment for interactions including *CD40LG-CD40, IL21-IL21R*, and *CD28-CD80* in the Low Responders (Fig. 5F). Thus, the functional deficit in Low Responders does not stem from a failure to express critical Tfh ligands or form receptor-ligand contacts, nor was it associated with loss of core Tfh transcriptional programs.

To further interrogate the transcriptional gene regulatory network of GNG4+Tfh, we first reconstructed the single-cell regulatory networks using gene regulatory network analysis via decoupleR (77). The regulatory landscape of High Responders was anchored by classical helper and maintenance transcription factors, including heightened activity of the *BCL6, SMAD2, CREM*, *STAT4*, and *MAF* regulons despite reduced gene expression of a select few (e.g. *POU2AF1*) (Fig. 5G, Supplemental Table 6). In contrast, the regulatory architecture of GNG4+Tfh cells in Low Responders was dominated by stress-induced and inflammatory transcription factors including *STAT1*, *RELA*, *NFκB1*, *IRF5*, and *CIITA*. We also performed SCENIC regulon analysis (64, 65) to evaluate specificity of the gene regulatory network differences (Fig. 5H, S5F), which also demonstrated greater specific regulon activity for *BCL6* and *MAF* in GNG4+Tfh from High Responders than from Low Responders. Furthermore, gene regulatory networks for AP-1 complex members such as JUN and FOS were notably enriched in the Tfh cells of Low Responders (Fig. S5F). These data implicate transcriptional network “noise” in the GNG4+Tfh from Low Responders as a potential mechanism for inability of the Tfh to provide adequate help, in spite of expression of *IL21*, *CD40LG*, *BCL6*, and other elements of the canonical Tfh transcriptional program.

To further interrogate the transcriptional networks that distinguished the Responder cohorts, we performed pathway analysis using the MSigDB Hallmark gene sets (78). Single-cell pathway analysis (SCPA) revealed a profound transcriptional rewiring of GNG4+Tfh cells in Low Responders (Fig. 5I). Strikingly, GNG4+Tfh cells from the Low Responders cohort displayed potent inflammatory and stress-response signatures, including “Interferon Alpha Response” and “Interferon Gamma Response” gene sets. In contrast, TNF-NFκB signaling was associated with High Responders’ GNG4+Tfh, underscoring a central role for NFκB signaling in Tfh help. GSEA demonstrated negative normalized enrichment score (NES) for both “TNFa Signaling via NFκB” (NES = −1.84, q-val = 2.7e-05) and “Interferon Alpha Response” (NES = −1.82, q-val = 1.6e-4), indicating massive upregulation of these pathways in the Low Responder Tfh cells (Fig. 5J–K). Similarly, the transitional GPR183 subset in Low Responders exhibited parallel dysregulation, with enrichment for hypoxia, apoptosis, and TGF-beta signaling pathways (Fig. S5G). Additionally, the TNFR2 subset demonstrated similar indicators of dysregulation with enrichment of hypoxia and TNFa/NFκB signaling pathways (Fig. S5H). Taken together, our results demonstrate that poor humoral responses to HBV vaccination are driven both by an inability to quantitatively expand the GNG4+Tfh pool and a qualitative, cell-intrinsic molecular dysfunction characterized by interferon-associated hyperactivation that impaired effective T-B intercellular communication within the GC.

## DISCUSSION

In this study, we combined longitudinal clinical profiling of HBV vaccine responses with ultrasound-guided lymph node biopsy to directly interrogate the GC reaction underlying variable human vaccine responsiveness. By pairing single-cell BCR and TCR repertoire analysis with transcriptomic and regulon profiling of B and T cell compartments from the same draining lymph nodes, we find that divergent antibody outcomes are not explained by a global failure of GC formation, convergent antigen recognition, or absence of T cell help. Rather, Low Responders mount a qualitatively and quantitatively distinct Tfh program: a contracted, transcriptionally rewired GNG4+ GC-Tfh population that retains some canonical helper features yet fails to sustain the costimulatory and gene-regulatory architecture required for productive LZ licensing. This suggests a specific, molecularly-defined Tfh defect at the center of suboptimal human vaccine responses.

A notable feature of the post-vaccination lymph node was the degree of BCR convergence we observed, both across unrelated participants in our cohort and with previously published HBV-specific sequences. Public or semi-public clonotypes have now been described across a range of human antibody responses, including dengue (79), SARS-CoV-2 (54), and HIV (80). Moreover, convergent BCR evolution has been described in influenza (81) and in other vaccination settings as well (82), and may reflect the constrained set of BCR that can recognize an immunodominant epitope. We found that convergence was concentrated in GC B cell and plasma cell states and was accompanied by high rates of *IGHV* mutation and IgG class-switching, which indicates that this is an antigen- and selection-driven phenomenon rather than an artifact of naive repertoire sharing. However, convergent clonotypic responses did not track with responder status in our cohort, as the recruitment of BCR specificities into the GC occurred even in individuals who ultimately mounted weak antibody titers. This argues that the principal bottleneck limiting humoral output in Low Responders lies downstream of antigen engagement and clonal recruitment rather than at the level of BCR repertoire availability. When considering vaccine design, strategies aimed purely at broadening initial antigen recognition such as via recruitment of diverse founder clones may be insufficient alone without also addressing the cellular processes that sustain GC selection.

Beyond repertoire-level convergence, transcriptomic profiling also revealed that High and Low Responders diverged sharply in GC B cell state. High-responder DZ and LZ B cells were enriched for heat-shock/co-chaperone programs supportive of BCL6 stability, together with MYC and mTORC1 activity consistent with active cyclic re-entry between LZ and DZ compartments. Low-Responder GC B cells, by contrast, showed features more consistent with premature extrafollicular commitment or B cell quiescence, including *JCHAIN*, *SELL*, *SYK*, and *FCRL1* expression, a signature associated with early activation or non-GC fates rather than sustained affinity maturation. This divergence was mirrored at the regulon level, where high-Responder LZ B cells were governed by *RELB/NFκB2* that can occur downstream of CD40 signaling, whereas Low Responder LZ B cells retained a core identity program including *BCL6* but without the active-selection signature. Together, these B cell-intrinsic findings indicate that Low Responders’ GCs adopt some features of canonical GC response but are unable to efficiently progress through iterative cycling in the GC, ultimately raising the question of the contribution of Tfh to this process. Indeed, we found that only a subcluster of Tfh, as defined by expression of *GNG4,* is localized to the GC and may represent a candidate cellular biomarker in the lymphoid tissue in humans. This subset was both quantitatively depleted and qualitatively altered in Low Responders, who showed persistent AP-1 activity (*JUN*/*FOS*), a regulon signature (*STAT1, RELA, NFκB1, IRF5, CIITA*) more typical of an inflammatory state than a helper program, and weakened/altered ligand-receptor connectivity with LZ B cells. Unexpectedly, GNG4+Tfh from Low Responders had greater expression of *IL21*, *CD40LG*, and *PDCD1*, which suggests these features were unable to overcome the dysfunction leading to poor humoral responses. Indeed, our regulon analysis shows greater BCL6 and cAMP-responsive element modulator alpha (*CREMα*) in the High Responders which are associated with productive GC reactions (83, 84). These findings extend the understanding that Tfh cells are not a monolithic population but comprise multiple transcriptionally and functionally distinct maturation states [refs], and that inflammatory microenvironments can skew Tfh differentiation toward non-productive states even when canonical markers are retained.

Our study has several limitations that should inform the design of future work. First, only a minority of participants had lymph node enlargement amenable to biopsy, and although we did not detect systematic associations between the likelihood of enlargement and available demographic or clinical metadata, we cannot fully exclude sampling bias in the CNBx subset, nor can we rule out that individuals lacking accessible lymphadenopathy differ systematically from those included. Second, our cross-sectional biopsy design captures a single time point (10–21 days post-vaccination) and therefore cannot distinguish whether the Tfh alterations we observe reflect delayed GC initiation, premature GC contraction, or a stable dysfunctional state sustained throughout the response; longitudinal sampling, for example by serial fine-needle aspiration, would help resolve the kinetics of GC and Tfh dysfunction and could reveal whether early inflammatory Tfh states are reversible. Third, our sample size, and in particular the small number of CpG-adjuvanted participants undergoing biopsy, limited our ability to formally test whether adjuvant choice modifies the cellular programs described here, despite the established clinical benefit of CpG adjuvants for HBV vaccine responses. Larger cohorts will be needed to determine adjuvant mechanisms and whether adjuvants can in part overcome Tfh dysfunction. Finally, our data establish a strong association between GNG4+Tfh and antibody outcome, but establishing causality will require additional functional and interventional studies.

Taken together, our data support a model in which productive humoral immunity requires coordinated engagement across both arms of the GC: LZ selection driven by non-canonical NF-κB signaling in B cells paired with an intact, spatially-restricted GNG4+Tfh population capable of delivering critical help. Although derived from HBV vaccination, the pathways implicated here, including non-canonical NF-κB/CD40 signaling, AP-1-driven inflammatory skewing, and interferon hyperactivation in Tfh, are not vaccine-specific, and parallel defects may underlie poor responses in other chronically inflamed populations, such as in the settings of aging and obesity. Whether Tfh dysfunction reflects a stable, individual-intrinsic trait or a state-dependent response to a given inflammatory context remains an open question that will be essential for designing future rational vaccination strategies.

## RESOURCE AVAILABILITY

### Lead contact

Inquiries for further information, data or resource availability may be directed to and fulfilled by lead contact and corresponding author, Ramin Herati (ramin.herati@nyulangone.org).

## METHODS

### Study design and sampling

This study was conducted as a prospective, open-label, partially-randomized evaluation of immunological outcomes of Hepatitis B virus (HBV) vaccination in adults (NCT04674462). Following approval by the NYU Institutional Review Board (protocol 20-01782), written informed consent was obtained from all participants prior to enrollment and study was carried out in accordance with the principles outlined in the Declaration of Helsinki. We recruited a total of 101 ambulatory participants stratified into two main experimental cohorts: a de novo vaccination cohort of HBV-naive adults (n=59) and a recall booster cohort of previously immunized, HBV-immune adults (n=42) based on history and medical chart review. One participant in the HBV-naive cohort was unable to comply with study procedures and was thus excluded from the remainder of the study. In the *de novo* cohort, vaccinations were administered in accordance with manufacturer package insert recommendations. To ensure balanced assignment, we used a permuted block randomization strategy with a block size of 10, yielding a 1:4 allocation ratio of 2 CpG-adjuvanted and 8 alum-adjuvanted vaccine recipients per block. Participants in the HBV-naive cohort received either a two-dose series of CpG-adjuvanted vaccine (Heplisav-B, n=13) administered at baseline and at least 4 weeks later, or a standard three-dose series of alum-adjuvanted vaccine (Engerix-B, n=45) administered at baseline, at least 4 weeks, and at least 24 weeks following enrollment. Participants in the HBV-immune cohort received a single booster dose of the alum-adjuvanted vaccine at study entry. Longitudinal peripheral blood sampling was collected at baseline (w0), 1 week after each vaccination (w1), and 4 weeks following each vaccination (w4).

Serum anti-HBsAg antibody titers were quantified using quantitative ELISA kit (XpressBio cat# WB2896) according to the manufacturer’s protocol. Based on the anti-HBV surface antigen titers (HBsAb) at the end of the study, participants were stratified into High Responders (>100 mIU/mL HBsAb) and low/poor Responders (<100 mIU/mL HBsAb).

### Blood and serum processing

Lymph node samples were mechanically dissociated and filtered via 70 um filter then cryopreserved in fetal bovine serum (FBS, Fisher) with 10% dimethyl sulfoxide (DMSO). Serum was collected using serum separator tubes per manufacturer instructions (SST, BD Biosciences). All aliquots were stored at −80C until use. Lymph node biopsy samples were thawed in RPMI (ThermoFisher) supplemented with 5 mM MgCl2 and 100 IU/mL DNase (Fisher).

### Axillary lymph node core needle biopsy

To gain a direct window into human germinal center dynamics at the active site of secondary lymphoid response, an optional lymph node sub-study was introduced via a protocol modification several months after study initiation. This optional procedure was offered to the next 86 active participants. Participants opting into this procedure underwent ultrasound screening for enlarged ipsilateral axillary lymph nodes between 10 to 21 days after their final immunization. Of the 86 individuals offered this procedure, 72 (83.7%) consented to screening, from whom 10 individuals (HBV-naive-Alum, n=3; HBV-naive-CpG, n=1; HBV-immune-Alum, n=6) presented with lymph nodes amenable for biopsy. Ultrasound-guided core needle biopsies (CNBx) were successfully performed using an 18-gauge needle, yielding one tissue core that was immediately fixed in formalin and embedded in paraffin for immunofluorescence assessment and up to 3 remaining cores that were mechanically dissociated to yield between 3e^5^ - 1e^6^ viable single cells. Single-cell lymphoid suspensions were cryopreserved as described above. Only grade I adverse events were identified during these procedures.

### Immunofluorescence staining

Deidentified tonsils were obtained from NYU Center for Biospecimen Research & Development (CBRD) to serve as GC size comparators. FFPE tissue sections (5 um thick) were mounted and deparaffinized with HistoChoice clearing agent (Fisher) followed by graded ethanol series. Antigen retrieval was performed under high pressure with Dako Target Retrieval solution (Agilent). Sections were first blocked with 5% animal serum, then stained with primary antibody cocktail, consisting of Ki67 (mouse anti-human, clone B56), PD-1 (rabbit anti-human, clone EPR4877), IgD (rat anti-human, clone W18340A), followed by a secondary stain of anti-mouse Cy5, anti-rat Alexa Fluor 488 and anti-rabbit Alexa Fluor 555. Coverslips were applied with ProLong Gold DAPI mountant and cured for at least 24 hours prior to imaging. Fluorescent images were acquired using a 10x objective on Keyence BZ-X810 fluorescence microscope.

### CITEseq

Samples were blocked with Fc receptor blocking solution (Biolegend) and FcR Blocking solution (Miltenyi), then stained for Totalseq-C human hashtag oligos and a panel of surface protein antibodies (Biolegend). Cells were pooled, loaded on Chromium GEM-X microfluidics chips and ran on Chromium X controller (10x Genomics). Surface protein, gene expression, and V(D)J immune receptor libraries were constructed with accompanying kits (10x Genomics) and prepared as instructed by the manufacturer. Libraries were quantified by qPCR (NEB Next Library Quantification, Illumina) and pooled for sequencing on the NovaSeq 6000 at the NYU Center for Genomics and Systems Biology core and the NovaSeq X at the NYU Langone Genome Technology Center core.

### Single cell RNA sequencing data processing

CITEseq paired-end FASTQ reads were processed with *cellranger multi* algorithm and aligned to reference human genome GRCh38 with Cellranger v8.0.1 (10x Genomics). Seurat was utilized for single cell library processing and downstream analysis. Quality control filters for cell exclusion included low and high UMI counts, and high mitochondrial counts, specific to each batch processed. HTO demultiplexing of genetically distinct individuals was conducted by CLR normalization (*NormalizeData*()), *HTODemux*(), and Single nucleotide polymorphism (SNPs) identification via Souporcell (85). Doublets were identified and removed with scDblFinder (v1.20.2), assuming a doublet rate of approximately 0.4% per 1000 cells. Additionally, cells with *CD3E* and *CD19* coexpression were removed. Integration was conducted between different 10x Genomics chip lanes and batches (including normalization with SCTransform, *SelectIntegrationFeatures*() with 5000 features, and *FindIntegrationAnchors*() encompassing the first 20 dimensions, rpca reduction, and 20 k.anchors), followed by subsequent Harmony (v.1.2.4) integration of subclustered cell populations. Celltype annotations were facilitated via Azimuth (v0.4.6).

The previously annotated lymph node CD4+ T cell compartment was subsetted for targeted analysis. To identify distinct T follicular helper maturation states, Tfh cells, defined by canonical expression of *CXCR5*, *BCL6*, *CXCL13*, and *PDCD1*, were isolated and subjected to unsupervised subclustering. Following scaling and normalization, principal component analysis (PCA) was performed, and the top principal components were used to construct a k-nearest neighbor (KNN) graph using the *FindNeighbors()* function (dims=1:30). Community detection was executed via the *FindClusters()* function (resolution=0.5), and dimensionality reduction was visualized using Uniform Manifold Approximation and Projection (UMAP). Cluster-defining marker genes were identified using the Wilcoxon rank-sum test to compare each subset against all other Tfh cells.

Differential expression analysis of the Tfh subsets was performed using *FindMarkers*() function in Seurat, followed by filtering for genes that had an average log2FC > 0.5, had p_adj <0.05, and was expressed in at least 20% of the cells from either of the comparator groups.

### B cell receptor repertoire analysis

V(D)J CITEseq processed files were annotated via IgBlast (v1.22.0) and further formatted using Change-O (v1.3.4) (86). Cellular barcodes with multiple heavy chain sequences were excluded. Following per participant Hamming distance clustering, a clonal threshold of 0.17 was established by the gaussian mixture model. Germline sequences were inferred using *createGermlines()* from package Dowser (v2.3), mapping sequences to IMGT human V(D)J references. Proportion IGHV nucleotide mutations relative to germline were calculated using *observedMutations()* from SHazaM (v1.3.1) (87, 88). Using Dowser, sequences were formatted with *formatClones()* and clonal trees generated with *getTree()* by the phylogenetic maximum likelihood (phangorn) method. Trees were visualized with ggtree (v3.10.1). To analyze global sequence convergence, all sequences were clustered by common heavy chain V and J gene, junction length, and junction similarity by Hamming distance threshold of 0.17 estimated by GMM. Germline divergence for each node was calculated using dowser’s *getDivergence()*, representing branch distance from the germline root. Maximum germline divergence per clone was defined as the largest such distance across all tips in the tree. Aggregate tree length was calculated as the sum of all branch lengths within a clonal tree, reflecting the total accumulated mutational distance across the lineage. The relationship between maximum germline divergence and aggregate tree length was modeled separately for each responder category using robust linear regression (Huber type M estimation using rlm function from MASS package), restricted to the range of aggregate tree lengths represented in both groups. The difference in slopes was assessed using a stratified bootstrap (resampling within each responder category, n = 2,000 resamples), with the 95% confidence interval for the slope difference.

### Pseudotime Trajectory Inference

To model the developmental progression of Tfh cells, unsupervised pseudotime trajectory inference was performed using the Slingshot package (v2.14.0). No root state was specifically designated. Cells were ordered along the inferred developmental continuum. The pseudotime metric was incorporated into the Seurat object.

### Differential Abundance and Clinical Correlation

For differential abundance analysis, *miloR* v.2.6 (89) was used using k=30, d=30 settings (89). For each of 1000 unique seeds (1:1000), 15% of the cells were sampled for the analysis, then measures of central tendency were calculated across all aggregated runs. The magnitude of compositional shifts was evaluated by calculating the log-fold change and determining Cohen’s *d* effect size for each Tfh subset relative to the antibody titer threshold.

### Module Scoring and Pathway Enrichment Analysis

To evaluate transcriptional pathways, cells were scored against the MSigDB Hallmark gene sets (e.g., “h.all.v2026.1.Hs.symbols.gmt”) to evaluate global pathway activity. First, the function *compare_pathways()* from the Single-Cell Pathway Analysis (SCPA) (v. 1.6.2) was performed using default parameters, and P values were adjusted via false discovery rate (FDR) correction. In addition, gene set enrichment analysis was performed using the fgsea package (v. 1.32.4) on the differentially-expressed genes per subset, as determined by *FindMarkers()*, and gene set variation analysis was conducted via the scGSVA package (v0.0.23) and UCell (v2.10.1).

### CD4 T Cell Clonal Analysis

Single-cell T cell receptor (scTCR-seq) data was integrated with the transcriptomic Seurat object utilizing the scRepertoire R package (v1.11.0). HBV-specific clonotypes (sourced from Immune Epitope Database and Tools) were mapped by cross-referencing CDR3 sequences. Clonal diversity within each Tfh subset was quantified by calculating Shannon’s entropy. Clonal expansion was categorized by frequency (e.g., single or medium expanded) and overlaid onto the Tfh UMAP embeddings to evaluate the relationship between transcriptional state and clonal burst size.

### Gene Regulatory Network Analysis

Gene regulatory networks were evaluated using *run_ulm()* from the decoupleR package (v 2.12.0) on the Tfh and GC B cell subsets, then results were compared across antibody titer groups using Wilcoxon test followed by FDR adjustment. For the regulatory network reference, we employed CollecTRI (90), a comprehensive meta-resource that integrates multiple databases into a directed regulatory network pruned for high-confidence transcription factor-target interactions. In addition, SCENIC regulatory analysis was independently performed using the SCENIC R package (v1.3.1), and was facilitated by the GENIE3 (1.28.0), RcisTarget (v1.26.0), and AUCell (v1.28.0) packages. *InitializeScenic*() utilized human species-specific motif databases. Genes were excluded based on low detection levels and/or cell presence (minCountsPerGene = 3% of total cells, minSamples = 1% of total cells).

### Cell-Cell Communication

Ligand-receptor interactions were calculated with LIANA (v0.1.14). Rankings were based on aggregate scores from applicable methods (CellPhoneDBv2, NATMI, Connectome, SingleCellSignalR, and iTALK). Significant interactions were defined with a cut-off of 0.05. Interaction network modeling was targeted specifically to evaluate interactions originating from Tfh sub-populations to LZ and DZ B cells. Aggregate_rank metric was used as the summary statistic as calculated by LIANA, where rank=0 would imply strongest enrichment and rank=1 would imply weakest enrichment. The Difference in Aggregate_rank for Low Responders and High Responders was used to determine relative enrichment.

### Visium HD analysis

Tonsil data was downloaded from a publicly-available dataset from a 10 μm-section of a fresh-frozen tonsil from a 21-year-old male using the Visium Human Transcriptome Probe Set v2.0, then aligned and mapped using Space Ranger v3.1.1 (Visium HD Spatial Gene Expression Library, Human Tonsil (Fresh Frozen), 10x Genomics, published 2024-09-17). The Loupe Browser (v9.0) was used to inspect the data. Spatial transcriptomic output aligned to high-resolution H&E images at a bin resolution of 8μm were used. To ensure precise pixel-to-bin mapping, we utilized the *Read10X_Image()* function in Seurat with the platform-specific scalefactors_json.json file. Data were normalized using SCTransform. Dimensionality reduction was performed via Principal Component Analysis then UMAP. Unsupervised spatial clustering, as previously performed by the vendor, was available as part of the dataset and imported into the Seurat object, yielding 16 distinct microanatomical regions (spatial clusters). To integrate the high-resolution Tfh subsets defined in the scRNA-seq analysis with the spatial data, we utilized the Seurat label transfer workflow. The scRNA-seq object containing the annotated Tfh maturation states served as the reference, while the Visium HD dataset was treated as the query. Transfer anchors were identified between the two datasets using the *FindTransferAnchors()* function, and Tfh subset labels (e.g., GNG4, GPR183, TNFR2, VIM) were projected onto the spatial bins via *TransferData()*.

### Statistics

Nonparametric tests were preferentially used throughout using two-tailed tests at α=0.05, unless otherwise indicated. Where paired analyses were performed, the unpaired data points were excluded. Outlier analysis was not performed and thus outliers were not excluded from the primary analyses. Not significant denoted as N.S. Genes were considered differentially expressed at a false discovery rate (FDR) threshold <= 0.05. Prism 9.0 and R v4.4 were used to perform statistical analyses.

## Supplementary Material

Supplement 1

Supplement 2

## Figures and Tables

**Figure 1.**
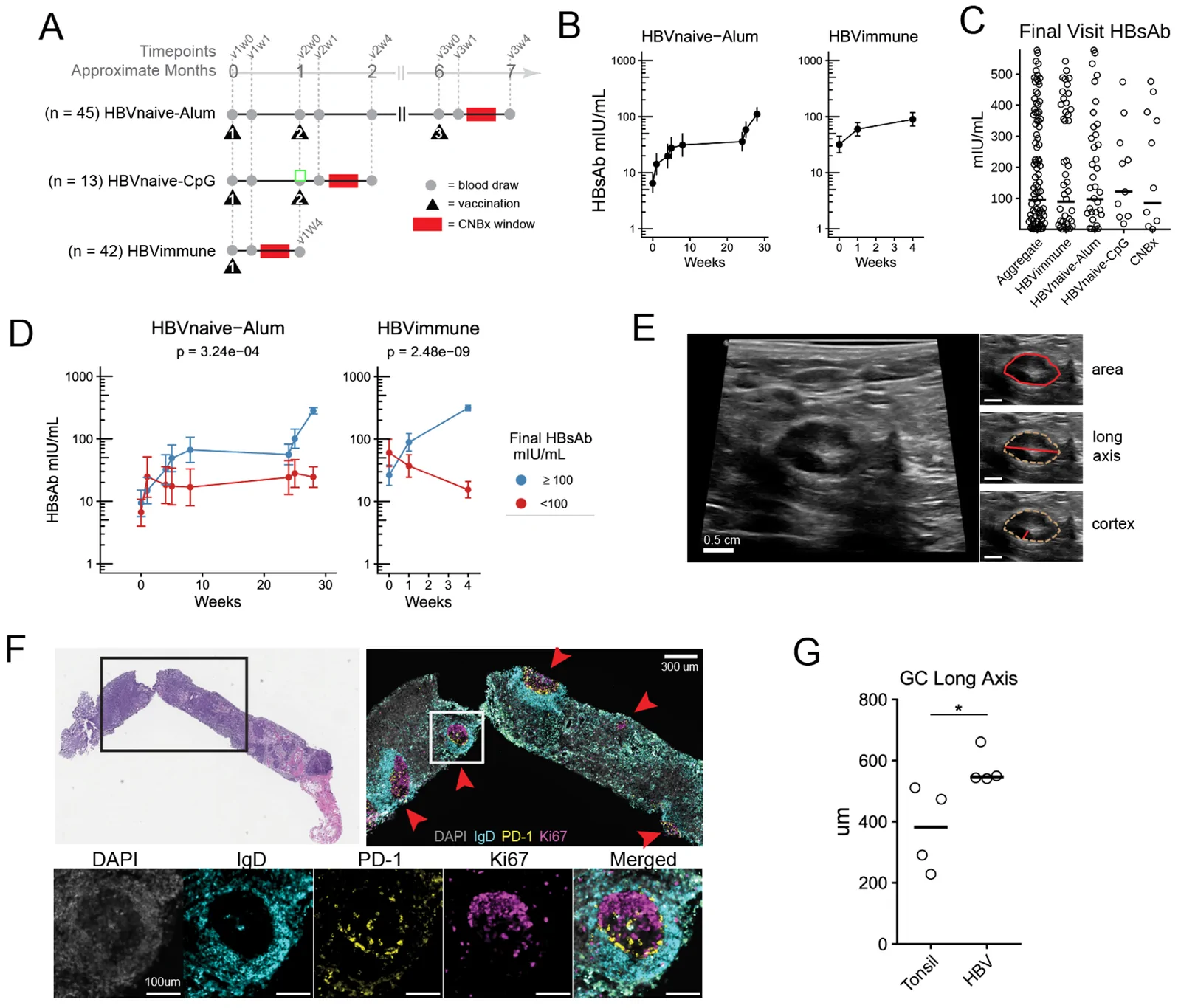
Heterogeneous humoral responses following Hepatitis B vaccination in adults **A**. Timeline of the clinical study showing vaccination schedules (black triangles), blood draw timepoints (gray circles), and core needle biopsy (CNBx) windows (red bars) across the HBVnaïve-Alum (n=45), HBVnaïve-CpG (n=13), and HBVimmune-Alum (n=42) cohorts. **B**. Longitudinal total HBsAb titers (mIU/mL) over time for the HBVnaïve-Alum and HBVimmune-Alum cohorts. **C**. Distribution of HBsAb titers at the final visit across the aggregate cohort, individual vaccine arms, and the subset of participants who underwent core needle biopsy (CNBx). Horizontal lines indicate medians. **D**. Longitudinal HBsAb kinetics stratified by responder status. Data shows geometric mean HBsAb titers +/− SEM over time for High Responders (blue) and Low Responders (red) for the HBVnaïve-Alum and HBVimmune cohorts. P-values indicate the group by time interactions by lme models on log transformed HBsAb, with Satterthwaite approximated degrees of freedom (lme4/lmerTest). **E**. Representative ultrasound imaging of an ipsilateral axillary lymph node post-vaccination. Insets detail cross-sectional area (red outline), long axis (orange dashed line), and cortical thickness (orange calipers). Scale bar = 0.5 cm. **F**. Top left: Low-power brightfield overview of the biopsy core. Top right: Low-power immunofluorescence for DAPI (cyan), IgD (gray), PD-1 (yellow), and Ki67 (magenta). Red arrowheads denote active germinal centers. Scale bar = 300 μm. Bottom: High-magnification focusing on a single germinal center. Scale bar = 100 μm. **G**. Quantitative comparison of germinal center long axis diameters (μm) between control human tonsil tissues (Tonsil GC) and CNBx (HBV GC). *P < 0.05. N.S., non-significant.

**Figure 2.**
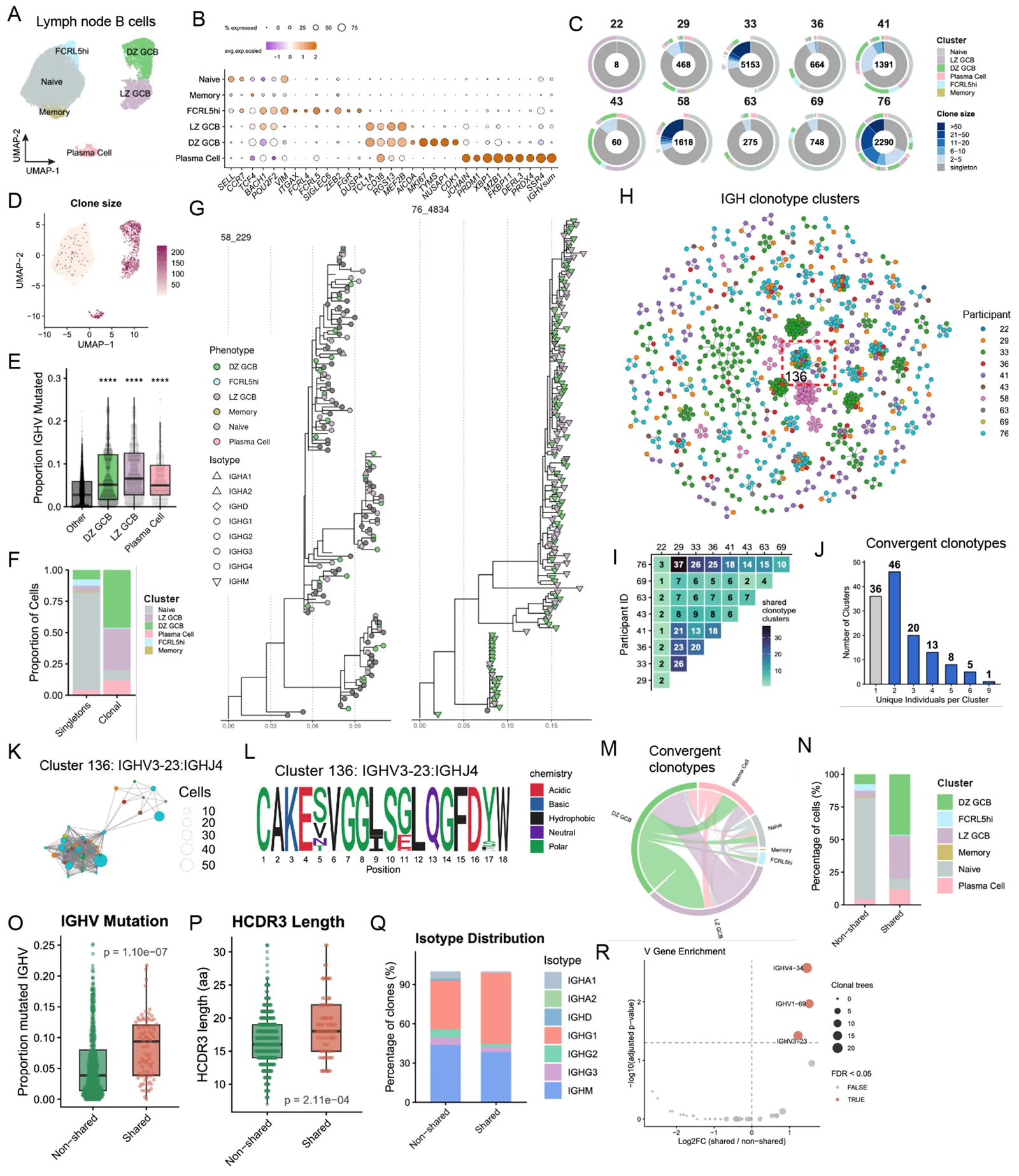
Lymph node biopsies reveal active and convergent germinal centers **A**. UMAP of core-needle lymph node biopsies (CNBx) for all participants. **B**. Expression of key lineage and functional marker genes across B cell subsets. **C**. Distribution of B cell phenotypes (outer ring) and clonal expansion size tiers (inner ring). Top number indicates participant and central number denotes total recovered BCR sequences. **D**. Distribution and density of expanded clones. **E**. Distribution of proportion mutated IGHV. Comparisons made relative to aggregated non GCB and PC clusters (denoted as Other). **F**. Proportion of B cell phenotypes compared between singleton (unexpanded) and clonal (expanded) BCR repertoires. **G**. Representative clonal lineage trees. Nodes are color-coded by cluster and shapes correspond to immunoglobulin heavy chain isotypes. **H**. IGH clonotype clusters across participants. Nodes colored by individual participant ID. Dashed red box denotes Cluster 136. **I**. Pairwise heatmap of exact number of shared convergent clonotype clusters between different participant pairs. **J**. Frequency distribution of convergent clonotype clusters. **K**. Expanded diagram of Cluster 136. **L**. Amino acid sequence logo of the conserved HCDR3 motif within Cluster 136. **M**. Clonotypic relationships between B cell subsets. **N**. B cell cluster frequencies between non-shared and shared clonotypes. **O**. Mean proportion of mutated IGHV nucleotides between non-shared and shared clonotypes (Wilcoxon P=1.2e-11). **P**. HCDR3 amino acid lengths for non-shared and shared clonotypes (P=2.1e-4). **Q**. Distribution of immunoglobulin heavy chain isotypes comparing non-shared and shared clonal sequences. **R**. Enrichment of specific IGHV genes (IGHV4-34, IGHV1-69, IGHV3-23) in shared versus non-shared repertoires.

**Figure 3.**
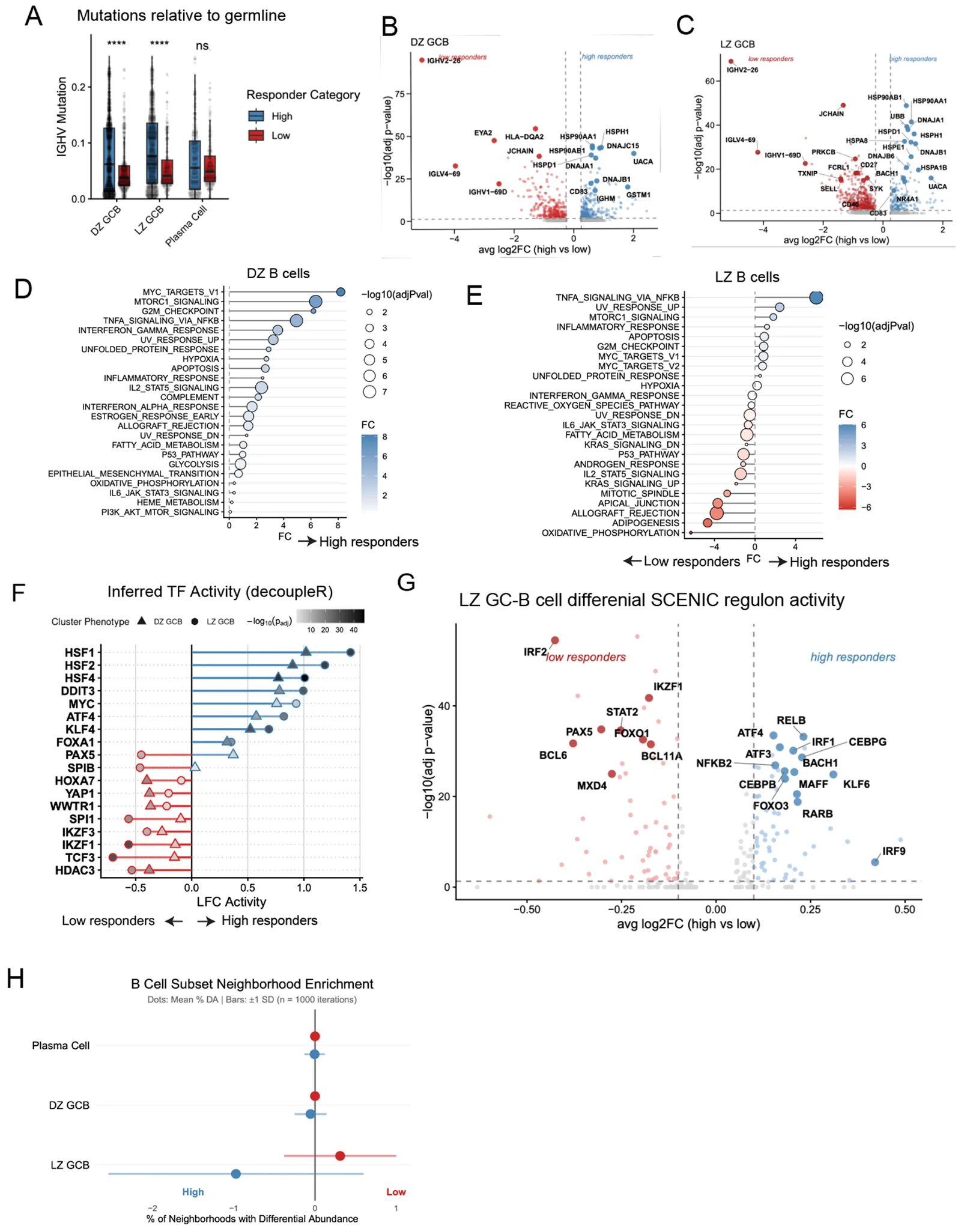
Germinal center B cell activity and transcriptional programs underlie robust humoral responses **A**. Somatic hypermutation frequencies (fraction of IGHV nucleotide mutations relative to germline) in light zone germinal center B cells (LZ GCB), dark zone germinal center B cells (DZ GCB), and plasma cells, stratified by High Responders (blue) and Low Responders (red). Wilcoxon rank-sum test (****p < 0.0001; ns, not significant). **B-C**. Differential gene expression between High and Low Responders within (B) dark zone germinal center B cells (DZ) and (C) light zone germinal center B cells (LZ). **D-E**. Single-cell pathway enrichment analysis for Hallmark pathways for High and Low Responders in (D) DZ B cells and (E) LZ B cells. **F**. Inferred gene regulatory network activity showing log-FoldChange (LFC) for DZ (triangles) and LZ (circles). **G**. Differential transcription factor regulon activity of LZ GCB by SCENIC analysis. **H**. Differential abundance (% DA) of cellular neighborhoods for Plasma Cells, DZ GCB, and LZ GCB cells stratified by Low (red) and High (blue) Responders. Dots indicate mean % DA; horizontal bars represent +/− 1 standard deviation calculated across 1,000 iterations.

**Figure 4.**
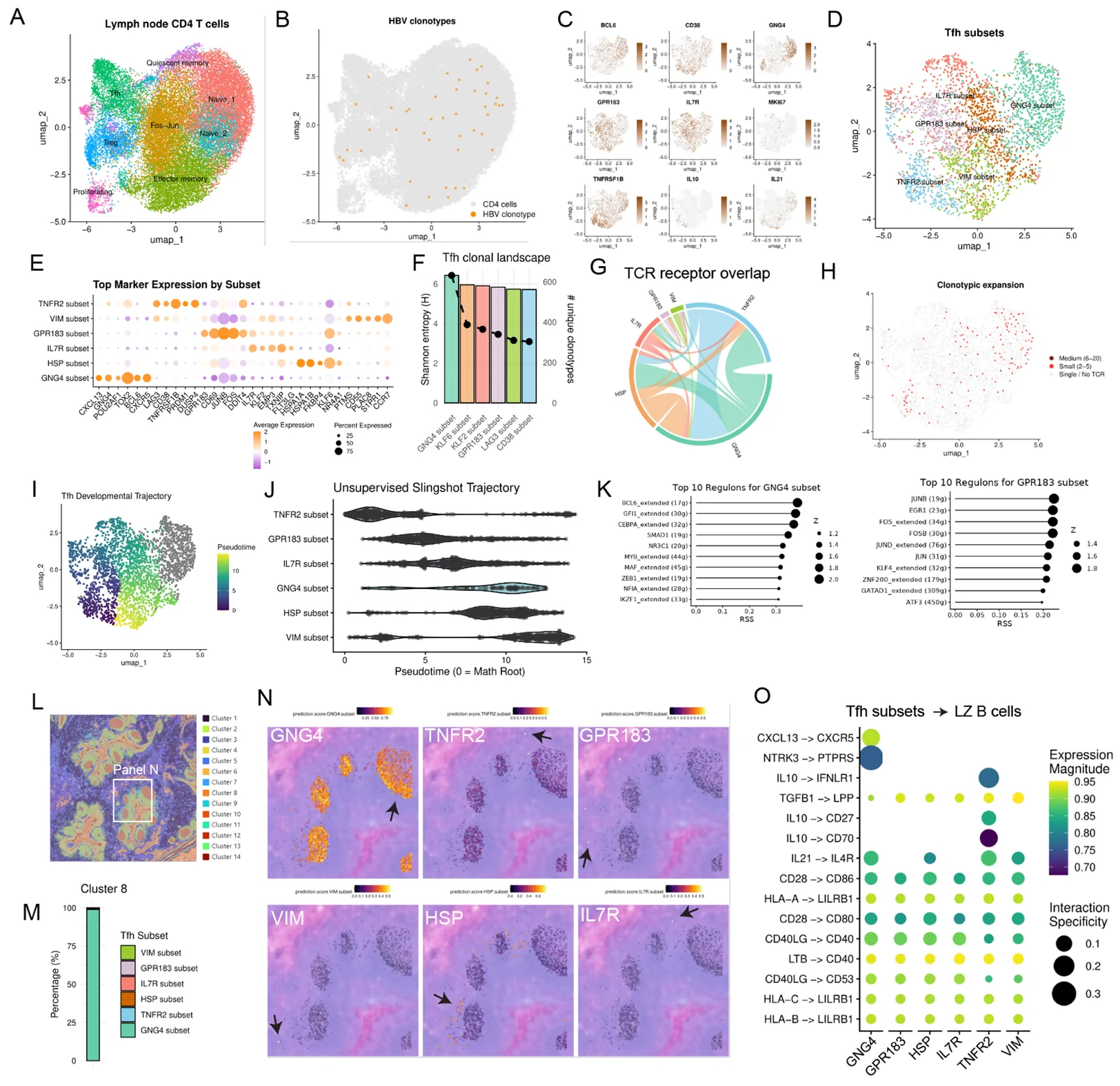
T follicular helper subsets are spatially segregated **A**. Total lymph node CD4+ T cells, colored by major phenotypic clusters. **B**. Distribution and distribution frequency of hepatitis B virus (HBV) specific T cell clonotypes within the CD4+ T cell landscape. **C**. Normalized expression profiles of key Tfh lineage and functional marker genes across the total Tfh population. **D**. Re-clustering of the Tfh population identifying six distinct sub-clusters: IL7R, GNG4, GPR183, TNFR2, VIM, and HSP subsets. **E**. Expression levels of top defining marker genes across the six Tfh subsets. Circle size denotes the percentage of cells expressing the gene, and the color scale represents the relative average expression level. **F**. Clonal diversity analysis of the Tfh landscape across sub-clusters, indicating Shannon entropy values (line, left y-axis) and the absolute number of unique clonotypes (bars, right y-axis). **G**. T-cell receptor (TCR) repertoire overlap and clonal sharing dynamics among the Tfh subsets. **H**. Clonotypic expansion within the Tfh population, categorized into Medium (6–20 cells), Small (2–5 cells), and Single/No TCR groups. **I**. Pseudotime trajectory map of Tfh development via Slingshot analysis overlaid on the UMAP space. **J**. Developmental distribution and density of individual Tfh subsets along the pseudotime trajectory line (0 = Math Root). **K**. Regulon specificity score (RSS) and Z-score rankings showing the top 10 master transcriptional regulons driving the GNG4 subset (left) and the GPR183 subset (right), predicted via SCENIC analysis. **L**. Spatial tissue architecture map across 14 distinct microanatomical clusters, with a black box delineating the region of interest (Panel N). **M**. Percentage composition and dominance of Tfh subsets within spatial Cluster 8. **N**. Spatial transcriptomic deconvolution and prediction score maps for the indicated Tfh subsets (GNG4, TNFR2, GPR183, VIM, HSP, and IL7R) within a representative lymph node tissue section. Black arrows indicate regions of specific spatial enrichment. **O**. Predicted ligand-receptor interactions between Tfh subsets (Source) and LZ B cells. Color intensity reflects the expression magnitude of the interaction pairs, and circle size denotes NATMI-derived interaction specificity.

**Figure 5.**
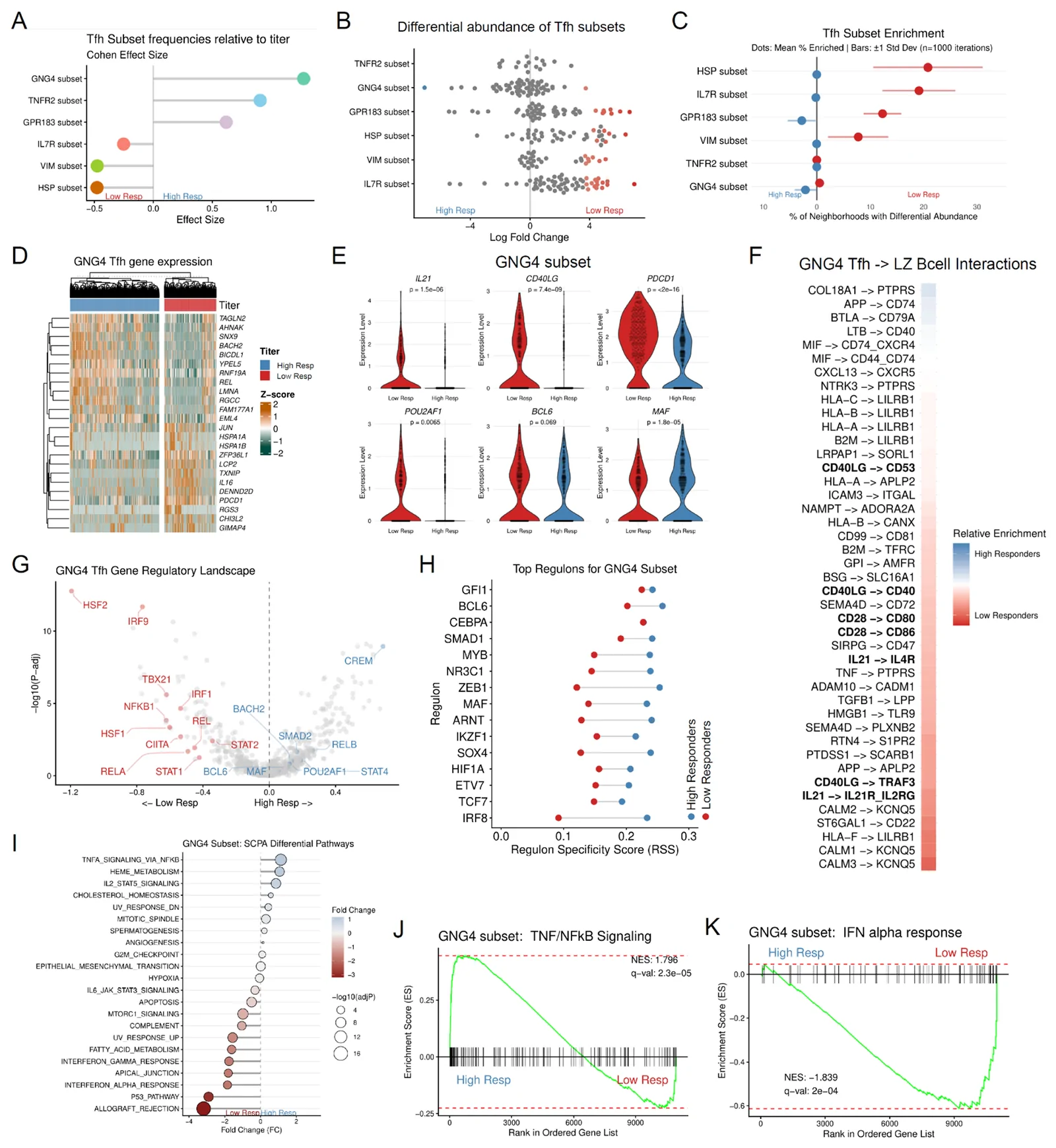
GNG4+Tfh cell expansion is a hallmark of robust humoral responses to HBV vaccination **A.** Cohen’s effect size for lymph node Tfh subset frequencies (GNG4, TNFR2, GPR183, IL7R, VIM, HSP) between High and Low Responders. **B**. Log fold-change distribution of major Tfh cell subsets across High versus Low Responder single-cell neighborhoods. **C**. Percentage of neighborhoods showing significant differential abundance. Dots represent mean percentage enriched, and bars indicate ±1 standard deviation across 1,000 iterations. **D**. Unsupervised hierarchical clustering of differentially expressed genes within the GNG4+Tfh subset, stratified by High and Low response groups. **E**. Single-cell expression for selected genes. P value by Wilcoxon test. **F**. Reciprocal ligand-receptor networks originating from GNG4+Tfh subset (source) targeting the LZ B cells (target). Relative enrichment represents the difference in enrichment scores from aggregate_rank as determined by LIANA. **G**. Differential gene regulatory network analysis of the GNG4+Tfh. **H**. Regulon Specificity Scores (RSS) for the top 10-ranked transcription factor networks governing the GNG4+Tfh subset across each of the High and Low response groups. **I**. Single-cell pathway analysis (SCPA) for differential activation of Hallmark pathways within the GNG4-subset between response groups. **J-K**. Gene set enrichment analysis profiles for the (**J**) TNF/NF-κB signaling and (**K**) IFN-α response pathways.

**Table:** KEY RESOURCES

REAGENT or RESOURCE	SOURCE	IDENTIFIER
**Antibodies**		
Anti-CD183 (clone G025H7, TotalSeq-C0140)	Biolegend	Cat# 126545
Anti-CXCR5 (clone J252D4, TotalSeq-C0144)	Biolegend	Cat# 356939
Anti-CD197 (clone G043H7, TotalSeq-C0148)	Biolegend	Cat# 353251
Anti-CD45RO (clone UCHL1, TotalSeq-C0087)	Biolegend	Cat# 304259
Anti-CD278 (clone C398.4A, TotalSeq-C0171)	Biolegend	Cat# 313553
Anti-CD279 (clone EH12.2H7, TotalSeq-C0088)	Biolegend	Cat# 329963
Isotype control IsoIgG1 (clone MOPC-21, TotalSeq-C0090)	Biolegend	Cat# 400187
Isotype control IsoIgG2A (clone MOPC-173, TotalSeq-C0091)	Biolegend	Cat# 400293
**Biological Samples**		
Lymph node biopsies and serum	NCT04674462	https://clinicaltrials.gov/study/NCT04674462?cond=NCT04674462&viewType=Card&rank=1
Tonsil tissue, deidentified	NYU Center for Biospecimen Resource and Development	
**Chemicals**		
ACK Lysis Buffer	ThermoFisher	Cat# A1049201
Human TruStain FcX (Fc Receptor Blocking Solution)	Biolegend	Cat# 422301
FcR blocking solution	Miltenyi	Cat# 130-059-901
Normal animal serum (blocking, 5%)		
HistoChoice Clearing Agent	Fisher	Cat# 50-199-030
Target Retrieval Solution	Agilent	Cat# GV80411-5
ProLong Gold Antifade Mountant with DAPI	Cell Signaling Technology	Cat# 8961S
**Equipment**		
Chromium X Controller	10x Genomics	
BZ-X810 microscope	Keyence	
**Consumables**		
70 um cell strainer	Fisher	Cat# 07-201-431
**Critical Commercial Assays**		
Anti-HBs quantitative ELISA kit	XpressBio	Cat# WB2896
NEBNext Library Quant Kit for Illumina	New England Biolabs	Cat# E7630S
Chromium Next GEM Single Cell 5′ Library Kit, v3	10x Genomics	Cat# 1000699
**Deposited Data**		
Single-cell RNAseq	This paper	GEO:____
Visium HD Spatial Gene Expression Library, Human Tonsil	10x Genomics	https://www.10xgenomics.com/datasets/visium-hd-cytassist-gene-expression-human-tonsil-fresh-frozen
HBV-specific T cell receptor sequences	IEDB	https://www.iedb.org/reference/1035279
**Software and Algorithms**		
R (v4.4.2)	R Foundation for Statistical Computing	https://www.r-project.org/
Cell Ranger multi (v8.0.1)	10x Genomics	https://www.10xgenomics.com/support/software/cell-ranger/latest
Seurat (v5.2.1)		Bioconductor
Souporcell (v2.5.0)		https://demultiplexing-doublet-detecting-docs.readthedocs.io/en/latest/Souporcell.html
scDblFinder (v1.20.2)		Bioconductor, RRID:SCR_022700
Azimuth (v0.4.6)		https://azimuth.hubmapconsortium.org
Harmony (v1.2.4)		https://github.com/immunogenomics/harmony
IgBlast (v1.22.0)		https://ncbi.github.io/igblast/
Change-O (v1.3.4)		https://changeo.readthedocs.io
Dowser (v2.3)		https://dowser.readthedocs.io
SHazaM (v1.3.1)		https://shazam.readthedocs.io
ggtree (v3.10.1)		Bioconductor
Slingshot (v2.14.0)		Bioconductor
miloR (v2.6)		Bioconductor
decoupleR (v2.12.0)		Bioconductor
CollecTRI		https://github.com/saezlab/CollecTRI
SCENIC (v1.3.1)		Bioconductor
GENIE3 (v1.28.0)		Bioconductor
RcisTarget (v1.26.0)		Bioconductor
AUCell (v1.28.0)		Bioconductor
SCPA (v1.6.2)		https://github.com/jackbibby1/SCPA
fgsea (v1.32.4)		Bioconductor
scGSVA (v0.0.23)		https://github.com/guokai8/scGSVA
UCell (v2.10.1)		Bioconductor
scRepertoire (v1.11.0)		Bioconductor
LIANA (v0.1.14)		https://github.com/saezlab/liana
Space Ranger (v3.1.1)	10x Genomics	https://www.10xgenomics.com
Loupe Browser (v9.0)	10x Genomics	https://www.10xgenomics.com
Custom bioinformatics scripts		doi: 10.5281/zenodo.2144076
