# Tfh dysfunction is associated with poor responses to HBV vaccination

**DOI:** 10.64898/2026.07.30.741822

**Published:** 2026-09-30

**Authors:** Han M. Chen, Elizabeth Severa, Xiaomin Yao, Tijaana Williams, Riddhimaa Sinha, Clarice Monteiro, Michael Tuen, Sam Barnett Dubensky, Sergei B. Koralov, Carla R. Nowosad, Derek A. Oldridge, Laura A. Vella, Anoma Nellore, Ramin Sedaghat Herati

## Abstract

The primary goal of vaccination is to induce durable protection through generating antigen-specific humoral immunity and cellular immune memory. Yet the same vaccine may elicit protection in some and not in others. To characterize the molecular and cellular events that underlie differential vaccine responsiveness, we leveraged the known, pronounced variability in vaccine antigen-specific antibody concentration and durability observed after Hepatitis B virus (HBV) immunization. We longitudinally profiled 101 healthy adults undergoing either *de novo* (n=59) or booster (n=42) HBV immunizations. We then stratified participants into High (≥100 mIU/mL) and Low (<100 mIU/mL) Responder groups based on end-of-study titers. From this cohort, we obtained core needle biopsies (CNBx) of vaccine-draining lymph nodes from 10 participants (High Responder n=6, Low Responder n=4), 10-21 days post-final vaccination and performed single-cell RNA sequencing with paired antigen receptor sequencing. Lymph node BCR repertoire analysis revealed convergent, semi-public clonotypes across unrelated participants with elevated somatic hypermutation. Transcriptomic profiling of GC-B cells uncovered functional divergence between response groups, with High Responders showing robust activation across both dark zone (DZ) and light zone (LZ) compartments and enrichment of MYC and mTORC1 signaling. This divergence in B cell responses corresponded to notable differences in the T cell compartment. High Responders harbored a key Tfh subset marked by increased expression of CXCL13, ICOS, and GNG4, supportive of their GC localization and capacity to provide B cell help. Low Responders demonstrated a divergent program in which their GNG4+Tfh displayed aberrant inflammatory transcriptional activity despite expressing coordinated costimulatory genes necessary to provide help to LZ GC-B cells. This inflammatory signaling was associated with reduced specificity of *BCL6* regulon activity despite maintained expression of canonical Tfh genes. Our data suggest that variable HBV vaccine responsiveness was associated with transcriptional aberrations in the GNG4+Tfh subset, offering a molecular framework for understanding poor vaccine responses.

**One Sentence Summary:**

Lymph node Tfh dysfunction is associated with poor vaccine responses to hepatitis B virus vaccination.

## Full Text Availability

The license terms selected by the author(s) for this preprint version do not permit archiving in PMC. The full text is available from the preprint server.

