# Supplementary material for: Tfh dysfunction underlies poor responses to HBV vaccination
Source: bioRxiv. 2026 Aug 4:2026.07.30.741822. Preprint. [Version 1] doi: 10.64898/2026.07.30.741822 (PMC13618653; doi:10.64898/2026.07.30.741822)
Supplement: Supplementary file 1 [file NIHPP2026.07.30.741822v1-supplement-2.pdf]

bioRxiv preprint doi: https://doi.org/10.64898/2026.07.30.741822; this version posted August 4, 2026. The copyright holder for this preprint (which was not certified by peer review) is the author/funder, who has granted bioRxiv a license to display the preprint in perpetuity. It is made available under a CC-BY 4.0 International license.

1066 # Supplemental Figures

bioRxiv preprint doi: https://doi.org/10.64898/2026.07.30.741822; this version posted August 4, 2026. The copyright holder for this preprint (which was not certified by peer review) is the author/funder, who has granted bioRxiv a license to display the preprint in perpetuity. It is made available under a CC-BY 4.0 International license.

Supplemental Figure 1

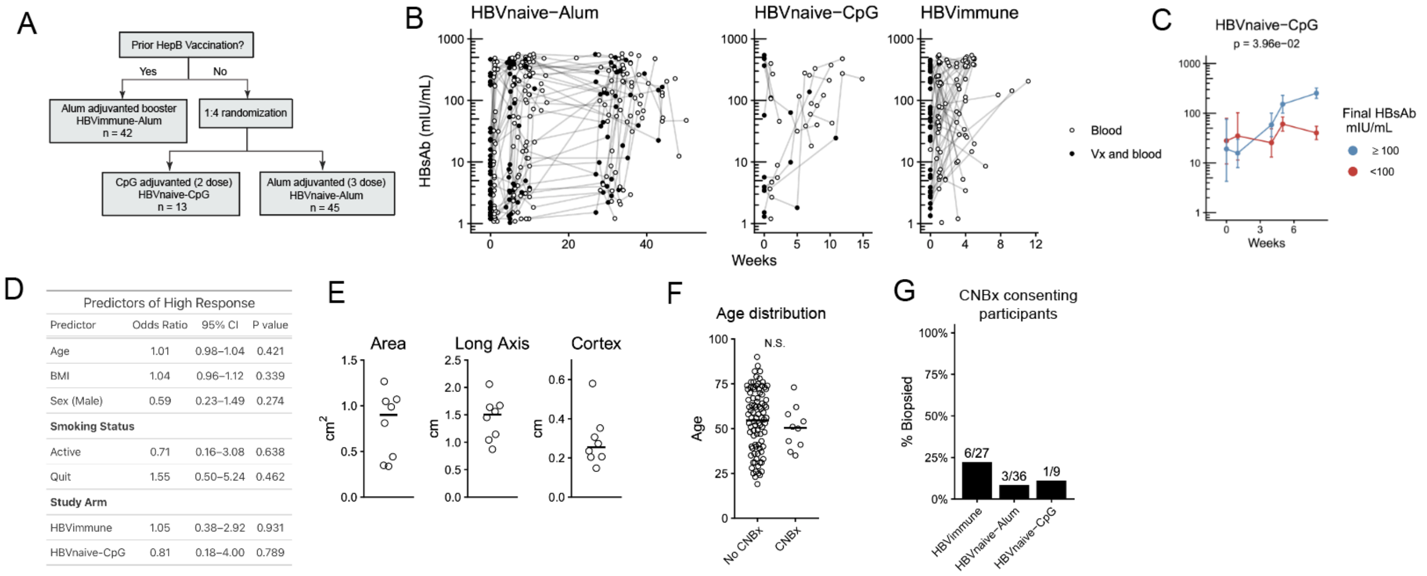

Predictors of High Response

Predictor	Odds Ratio	95% CI	P value
Age	1.01	0.98–1.04	0.421
BMI	1.04	0.96–1.12	0.339
Sex (Male)	0.59	0.23–1.49	0.274
**Smoking Status**			
Active	0.71	0.16–3.08	0.638
Quit	1.55	0.50–5.24	0.462
**Study Arm**			
HBVimmune	1.05	0.38–2.92	0.931
HBVnaive-CpG	0.81	0.18–4.00	0.789

Supplemental Figure 1. A. Participant randomization and cohort workflow. Individuals with prior vaccination received an alum-adjuvanted booster (HBVimmune-Alum, n=42). Vaccine-naive individuals underwent a 1:4 randomization to receive either a 2-dose CpG-adjuvanted vaccine (HBVnaive-CpG, n=13) or a 3-dose alum-adjuvanted vaccine (HBVnaive-Alum, n = 45). **B**. Longitudinal HBsAb titers by cohort. Individual longitudinal hepatitis B surface antibody (HBsAb) titers (mIU/mL) over time (weeks) across the three study cohorts. White circles indicate blood collection timepoints; black circles indicate simultaneous vaccination (Vx) and blood collection events. **C**. Longitudinal HBsAb kinetics stratified by responder status in HBVnaive-CpG. Data shows geometric mean HBsAb titers +/- SEM over time for High Responders (blue) and Low Responders (red). P-values indicate the group by time interactions by linear mixed effects models on log transformed HBsAb, with Satterthwaite approximated degrees of freedom (lme4/lmerTest). **D**. Multivariable logistic regression table for clinical and demographic predictors of achieving a high endpoint HBsAb titer. Model adjusts for Age, Sex (ref: F), Smoking Status (ref. Never), and Study arm (ref. HBVnaive-Alum). **E**. Lymph node anatomical measurements. Quantitative metrics of regional lymph nodes, including cross-sectional area, long axis length, and cortical thickness. Data points represent independent participants, with horizontal bars indicating the mean values. **F**. Participant ages for all participants and the subset undergoing CNBx. **G**. Proportion of participants who successfully underwent CNBx out of the total number screened.

bioRxiv preprint doi: https://doi.org/10.64898/2026.07.30.741822; this version posted August 4, 2026. The copyright holder for this preprint (which was not certified by peer review) is the author/funder, who has granted bioRxiv a license to display the preprint in perpetuity. It is made available under a CC-BY 4.0 International license.

Supplemental Figure 2

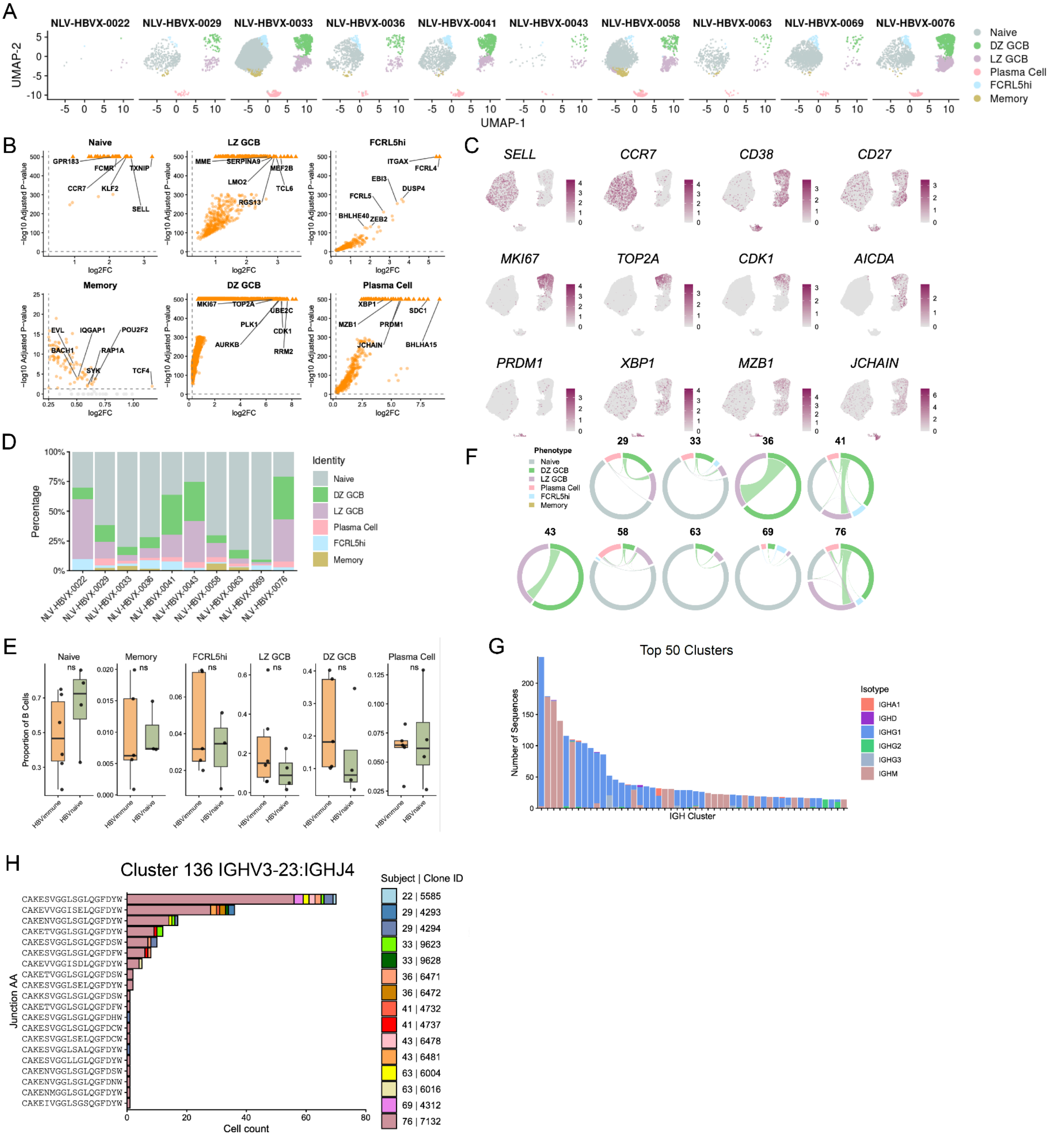

Supplemental Figure 2. A. Single-cell UMAP projections of sorted B cells from individual participants color-coded by identified B cell phenotypes: Naive, Dark Zone Germinal Center B cells (DZ GCB), Light Zone Germinal Center B cells (LZ GCB), Plasma Cell, FCRL5hi, and Memory B cells. **B**. Differentially expressed genes across each B cell subset. **C**. Expression of the normalized expression levels of individual marker

bioRxiv preprint doi: https://doi.org/10.64898/2026.07.30.741822; this version posted August 4, 2026. The copyright holder for this preprint (which was not certified by peer review) is the author/funder, who has granted bioRxiv a license to display the preprint in perpetuity. It is made available under a CC-BY 4.0 International license.

genes (SELL, CCR7, CD38, CD27, MKI67, TOP2A, CDK1, AICDA, PRDM1, XBP1, MZB1, and JCHAIN). **D**. Stacked bar plot showing the percentage distribution of the six B cell phenotypes within each individual participant. **E**. Proportions of each B cell phenotype between HBV-immune and HBV-naive cohorts. ns, not significant. **F**. Clonal distribution and phenotypic transitions across B cell subsets within individual participants. **G**. Total sequence counts and isotype composition for the top 50 expanded IGH clusters. **H**. Participant and clonal composition of IGHV3-23:IGHJ4 (Cluster 136) for unique HCDR3 amino acid sequences.

bioRxiv preprint doi: https://doi.org/10.64898/2026.07.30.741822; this version posted August 4, 2026. The copyright holder for this preprint (which was not certified by peer review) is the author/funder, who has granted bioRxiv a license to display the preprint in perpetuity. It is made available under a CC-BY 4.0 International license.

Supplemental Figure 3

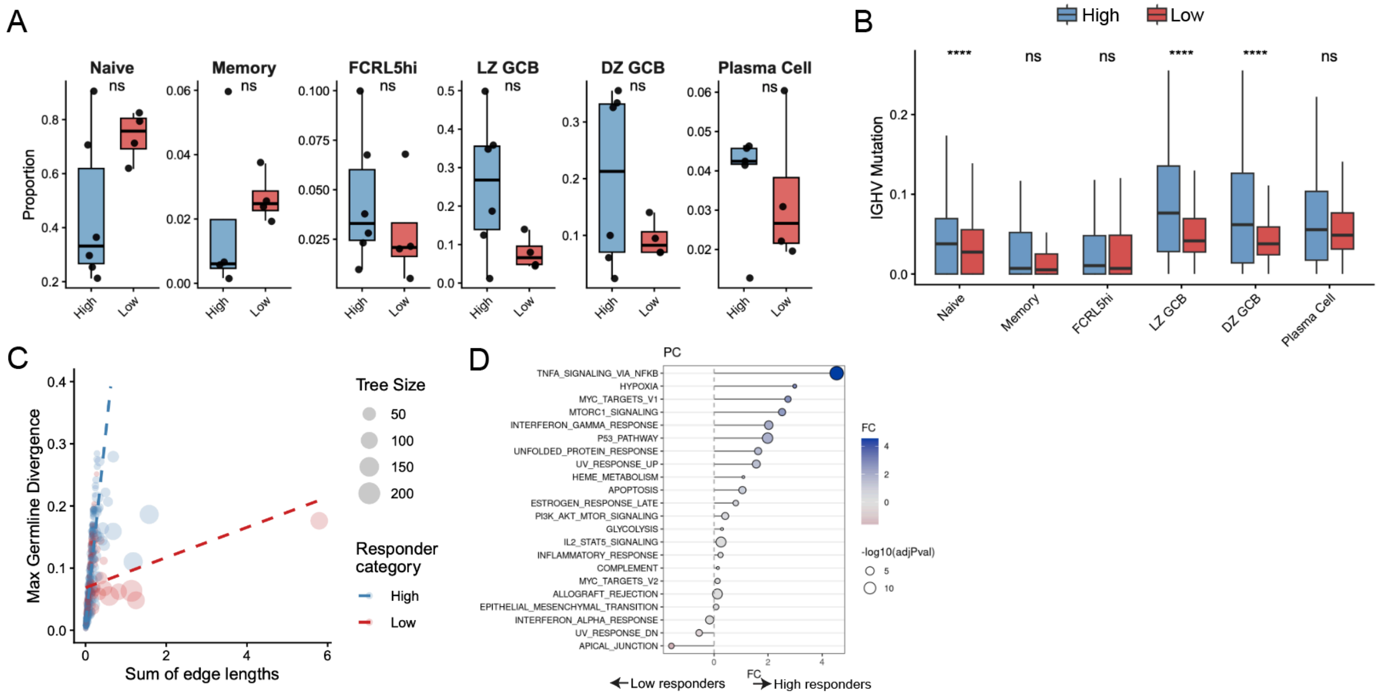

Supplemental Figure 3. **A**. Relative abundance of Naive, Memory, FCRL5hi, Light Zone Germinal Center B (LZ GCB), Dark Zone Germinal Center B (DZ GCB), and Plasma Cells between High and Low Responder groups. P value by Wilcoxon test. **B**. IGHV somatic hypermutation (SHM) loads across the designated B cell subsets between Over100 and Under100 cohorts. (****p < 0.0001, ns: not significant). **C**. Relationship between maximum germline divergence and aggregate tree length by responder category (robust linear regression, stratified bootstrap resampling, n = 2,000; slope difference = 0.469, P = 0.031). **D**. Differentially enriched Hallmark pathways in plasma cells (PC) between Low and High Responders.

bioRxiv preprint doi: https://doi.org/10.64898/2026.07.30.741822; this version posted August 4, 2026. The copyright holder for this preprint (which was not certified by peer review) is the author/funder, who has granted bioRxiv a license to display the preprint in perpetuity. It is made available under a CC-BY 4.0 International license.

1106 **Supplemental Figure 4**

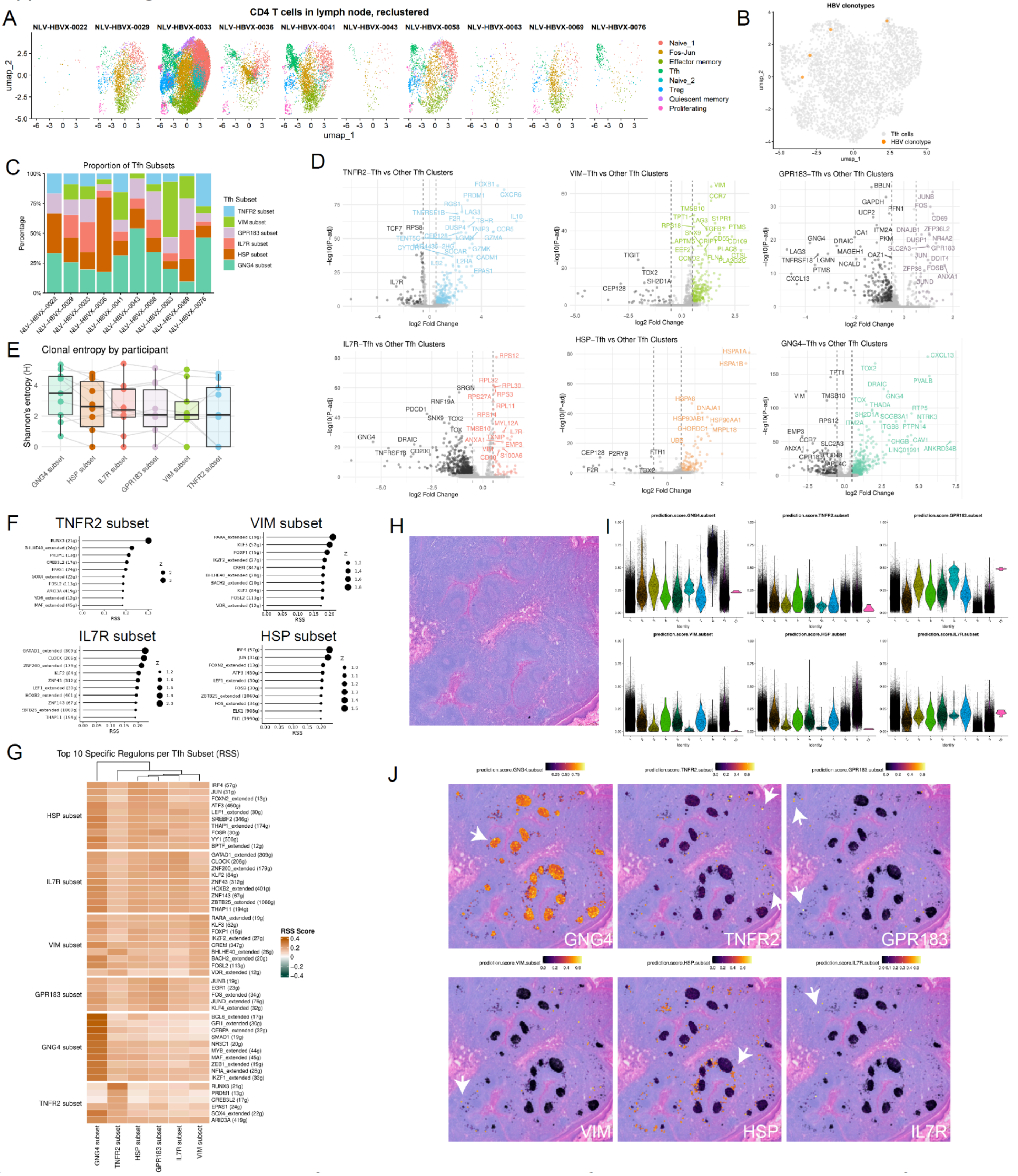

1107

bioRxiv preprint doi: https://doi.org/10.64898/2026.07.30.741822; this version posted August 4, 2026. The copyright holder for this preprint (which was not certified by peer review) is the author/funder, who has granted bioRxiv a license to display the preprint in perpetuity. It is made available under a CC-BY 4.0 International license.

Supplemental Figure 4. **A**. Reclustered CD4+ T cell UMAPs from ten HBV CNBx participants, colored by identified CD4+ T cell clusters. **B**. HBV-specific T cell clonotypes from literature overlaid onto the Tfh cell cluster. **C**. Distribution of six Tfh subsets across participants. **D**. Differential gene expression for each Tfh subset compared against all other Tfh clusters. **E**. Shannon's entropy of T cell receptor (TCR) clones within each Tfh subset stratified by participant. **F**. Top 10 specific regulons ranked by Regulon Specificity Score (RSS) for Tfh subsets. **G**. Unsupervised hierarchical clustering of the top 10 specific regulons and their respective RSS scores across Tfh subsets. **H**. Hematoxylin and eosin (H&E) stained section of tonsil used for spatial transcriptomics. **I**. Distribution of transfer scores for each Tfh subset across spatial tissue spots. **J**. Predicted transfer score for each Tfh subset overlaid onto the tissue architecture. White arrows indicate bins with high enrichment.

bioRxiv preprint doi: https://doi.org/10.64898/2026.07.30.741822; this version posted August 4, 2026. The copyright holder for this preprint (which was not certified by peer review) is the author/funder, who has granted bioRxiv a license to display the preprint in perpetuity. It is made available under a CC-BY 4.0 International license.

Supplemental Figure 5

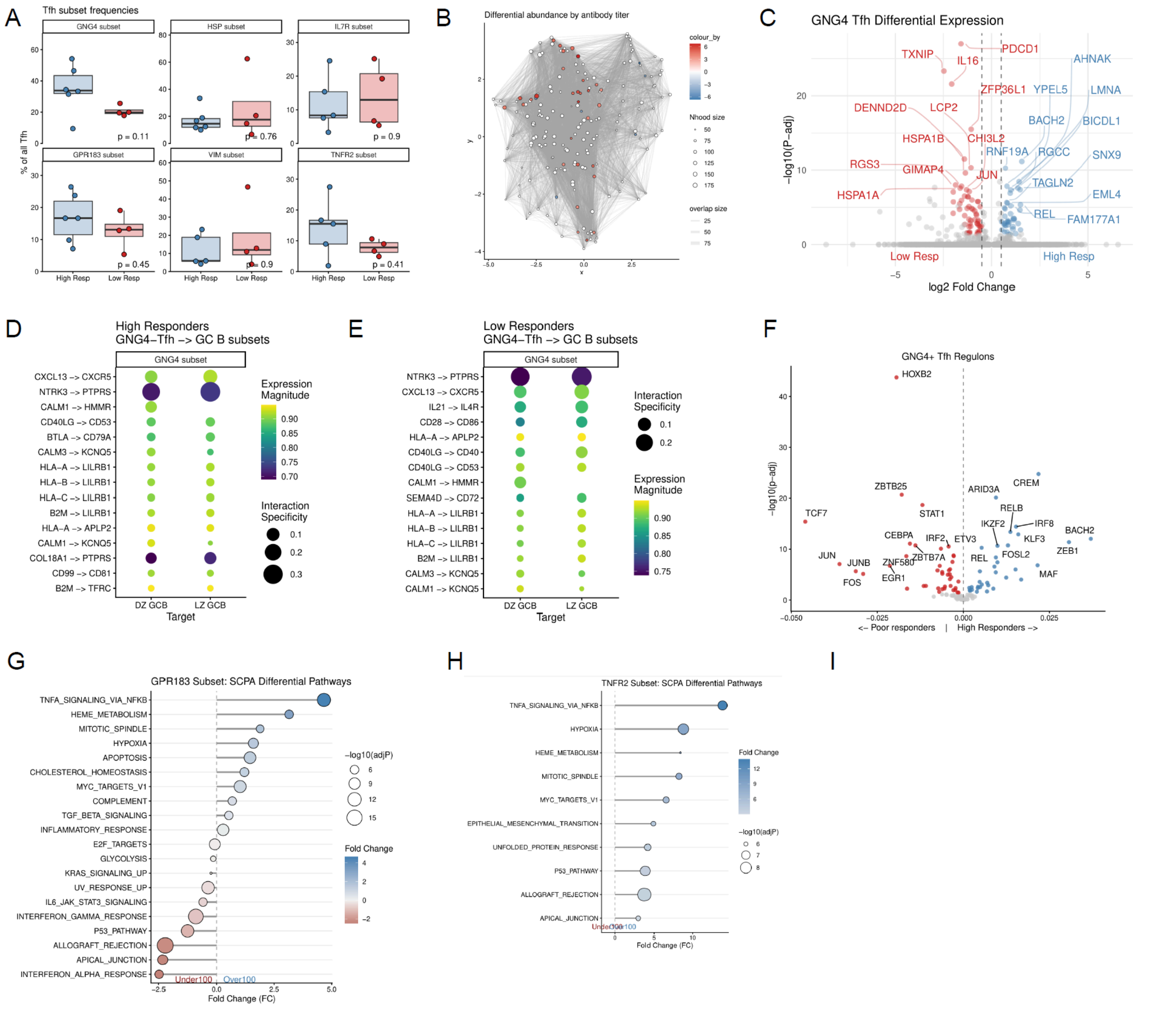

Supplemental Figure 5. A. Frequency of six Tfh subsets (as percentage of total Tfh cells) between High Responders and Low Responders. P-values by Wilcoxon test. **B**. Neighborhood network graph of differential cell abundance associated with antibody titers. Nodes represent cell neighborhoods, colored by log fold change (red indicates enrichment in Low Responders and blue indicates enrichment in High Responders). **C**. GNG4+Tfh differential expression between Low Responders (red) and High Responders (blue). **D-E.** Predicted ligand-receptor interactions originating from the GNG4+Tfh subset targeting Dark Zone (DZ) and Light Zone (LZ) Germinal Center (GC) B cells in (**D**) High Responders and (**E**) Low Responders. Circle size indicates NATMI-based interaction specificity, and the color gradient represents expression magnitude. **F**. Differential transcription factor regulon activity between Low Responders (red) and High Responders (blue). **G-H**. Single-Cell Pathway Analysis (SCPA) differential MSigDB Hallmark pathways for the (**G**) GPR183 subset

bioRxiv preprint doi: https://doi.org/10.64898/2026.07.30.741822; this version posted August 4, 2026. The copyright holder for this preprint (which was not certified by peer review) is the author/funder, who has granted bioRxiv a license to display the preprint in perpetuity. It is made available under a CC-BY 4.0 International license.

and (**H**) TNFR2 subset. Pathways are ranked by fold change.
